# Comparative Effectiveness of Rehabilitation Programs for Adults with Post-Tuberculosis Lung Disease: A Systematic Review and Meta-Analysis of Randomized Controlled Trials

**DOI:** 10.3390/jcm15218397

**Published:** 2026-10-28

**Authors:** Marin Vlăduț Piciorea, Gabriela-Marina Andrei, Mihai Olteanu, Daniela Matei, Mihaela Ionescu, Magdalena Rodica Trăistaru

**Affiliations:** 1 Doctoral School, University of Medicine and Pharmacy of Craiova, 2 Petru Rareș Street, 200349 Craiova, Romania; marin.piciorea@umfcv.ro; 2 Internal Medicine—Department of Pulmonology, University of Medicine and Pharmacy of Craiova, 2 Petru Rareș Street, 200349 Craiova, Romania; gabriela.andrei@umfcv.ro (G.-M.A.); mihai.olteanu@umfcv.ro (M.O.); 3 Department of Physiotherapy, Faculty of Nursing, University of Medicine and Pharmacy of Craiova, 2 Petru Rareș Street, 200349 Craiova, Romania; rodica.traistaru@umfcv.ro; 4 Department of Medical Informatics and Biostatistics, Faculty of Dentistry, University of Medicine and Pharmacy of Craiova, 2 Petru Rareș Street, 200349 Craiova, Romania; mihaela.ionescu@umfcv.ro

**Keywords:** post-tuberculosis lung disease, pulmonary rehabilitation, exercise capacity, six-minute walk test, telerehabilitation, randomized controlled trials, meta-analysis

## Abstract

**Background:** Persistent respiratory and functional impairments after pulmonary tuberculosis can reduce exercise capacity and health-related quality of life. This systematic review and meta-analysis evaluated structured rehabilitation in adults with post-tuberculosis lung disease (PTLD). **Methods:** The review followed PRISMA 2020 and was prospectively registered in PROSPERO (CRD420261338253). PubMed/MEDLINE, Scopus, Web of Science Core Collection, CENTRAL, and PEDro were searched from inception, with final bibliographic updating through 5 September 2026 and trial registry cross-linking through 28 August 2026. **Results:** Seven randomized trial reports were included. Two directly comparable adjusted ISWT trials are presented as the main exercise capacity findings: a study published in 2026 reported +54.36 m (95% CI 17.22 to 91.51) and a research conducted in 2026 reported +123.0 m (95% CI 81.1 to 164.8). The pooled random-effects estimate was +87.98 m (95% Hartung–Knapp CI −348.01 to 523.96), but this estimate is presented secondarily because the interval was extremely wide with only two contributing trials. A 120-participant RCT reported an adjusted 6MWD benefit of +87.4 m (95% CI 74.2 to 100.6). A newly identified three-arm RCT (*n* = 180) reported statistically significant omnibus between-group differences, but substantial baseline imbalance and unusual control group reporting patterns limit device-versus-control interpretation. Certainty was very low for exercise capacity, dyspnea, health-related quality of life, pulmonary function, inspiratory muscle strength, and adverse events. **Conclusions:** Accordingly, the evidence is very uncertain regarding the effects of structured rehabilitation on these outcomes and regarding the magnitude and generalizability of any benefit in PTLD.

## 1. Introduction

Although tremendous progress has been made in the prevention, diagnosis, and treatment of tuberculosis (TB), it continues to be a major global health problem. According to the World Health Organization Global TB Report 2025, an estimated 10.7 million people developed tuberculosis worldwide in 2024, 1.23 million people died from TB, and the global incidence rate was 131 cases per 100,000 population [1]. However, successful antimicrobial treatment does not always mean full lung recovery. Patients who have been treated for pulmonary TB may still experience residual lung function abnormalities, impairment of health-related quality of life, fatigue, loss of working capacity, and reduced physical activity. Post-tuberculosis lung disease (PTLD) is the term that refers to chronic respiratory dysfunction with or without symptoms that occur due to TB. PTLD includes various conditions, such as abnormal lung function tests, restriction or obstruction of the airway, gas exchange abnormalities, respiratory and peripheral muscle dysfunction, and physical deconditioning [2]. According to a study that analyzed the prevalence of post-treatment respiratory failure after pulmonary TB, 59.1% of participants had abnormal spirometry findings [3]. Thus, PTLD can have a significant impact on a person’s health-related quality of life, functional performance, and healthcare resource use.

Pulmonary rehabilitation is an individualized intervention that includes patient-centered strategies, such as exercise training and education, leading to improved physical activity levels and enhanced well-being. It also involves nutritional and psychological counseling and is considered a comprehensive approach to managing patients with PTLD. International guidelines on the management of PTLD recommend screening for continued post-TB impairment and considering pulmonary rehabilitation for those with persistent symptoms or functional limitations [3]. The possible benefits of pulmonary rehabilitation include improved exercise tolerance and muscle strength, reduced dyspnea and fatigue, increased functional capacity, and improved health-related quality of life [2]. Nevertheless, the interventions that have been undertaken in patients with PTLD varied greatly in terms of interventions, intensity, duration, exercise prescription, supervision, setting, and additional components, and the evidence mostly comes from small studies or non-randomized trials.

Recent evidence syntheses have examined the potential benefits of pulmonary rehabilitation in patients with post-tuberculosis lung disease (PTLD). Gebremedhn et al. included 13 studies with 747 participants: one randomized controlled trial, one prospective-cohort study, and 11 pre–post interventional studies. In their pooled analysis, they found a mean within-group improvement of 60.04 m in the six-minute walk test, but evidence certainty for this finding was very low [4]. More recently, Kumar et al. published a systematic review with 17 studies of randomized and observational design, in which they found largely favorable findings for exercise capacity and health-related quality of life, while pulmonary function findings were heterogeneous; the authors also emphasized the need for future high-quality trials [5]. Since both reviews included non-randomized or uncontrolled designs, and since within-group change cannot differentiate an intervention effect from spontaneous recovery, regression to the mean, repeated testing, patient motivation, or confounding concurrent influences, the magnitude of the comparative effect uniquely attributable to rehabilitation remains uncertain.

The main evidence gap, therefore, is not whether rehabilitation is associated with improvement within treated groups, but whether it produces benefits in excess of those observed under a concurrent control condition and whether those benefits are supported by sufficiently rigorous randomized evidence. The present review therefore complements the recent broader evidence syntheses by restricting eligibility to randomized controlled trials and focusing on comparative between-group effects. It reduces confounding and the risk that uncontrolled within-group improvement is interpreted as an intervention effect. The aim of this systematic review and meta-analysis was to evaluate the comparative effectiveness and safety of structured rehabilitation programs for adults with PTLD. The primary outcome was exercise capacity immediately after completion of the intervention. Secondary outcomes included pulmonary function, dyspnea, health-related quality of life, muscle strength, adverse events, hospitalizations, and respiratory exacerbations. Risk of bias was assessed using the Cochrane RoB 2 tool, and certainty of evidence was evaluated using GRADE.

## 2. Materials and Methods

### 2.1. Protocol Registration and Reporting Standards

This systematic review and meta-analysis followed the methodological recommendations in the Cochrane Handbook for Systematic Reviews of Interventions and adhered to the PRISMA 2020 statement; the literature search was informed by the PRISMA-S extension [6,7,8]. The protocol was registered in the International Prospective Register of Systematic Reviews (PROSPERO; CRD420261338253) on 12 March 2026. The review question, eligibility criteria, outcomes, and overall synthesis plan were specified before the study selection. The protocol specified searches of Embase and CINAHL; these databases were inaccessible because of the absence of institutional subscriptions. Reference-list screening was retained as a means of supplementary identification. No changes were made to the target population, intervention, comparator, primary outcome, or restriction to randomized controlled trials. 

During the preparation of this manuscript, OpenAI was used to assist with language editing, textual verification, and editorial refinement. All AI-assisted suggestions were critically reviewed by the authors, who take full responsibility for the final manuscript content.

### 2.2. Eligibility Criteria

Eligibility was defined according to the Population, Intervention, Comparator, Outcomes, and Study design (PICOS) framework; the outcomes are detailed in Section 2.5.

Population. Eligible studies enrolled adults aged 18 years or older with a history of pulmonary tuberculosis who have completed anti-tuberculosis treatment but had persistent respiratory symptoms and/or residual structural, physiological, or functional impairment attributable to previous pulmonary tuberculosis, as defined by the original trial. Eligible PTLD definitions could include persistent symptoms, abnormal chest imaging, spirometric impairment, exercise limitation, or combinations of these features. Participants with active pulmonary tuberculosis were excluded. No minimum interval beyond completion of anti-tuberculosis treatment was imposed unless specified by the individual trial. Studies of mixed populations were eligible only when data for participants with PTLD could be extracted separately. Studies confined to extrapulmonary tuberculosis without pulmonary sequelae were excluded.

Intervention. Eligible interventions were structured rehabilitation programs containing a planned exercise or respiratory-training component. This could include aerobic or endurance exercise, resistance training, respiratory muscle training, breathing exercises, or combinations of these modalities. Programs could be supervised, partially supervised, home-based, or delivered through telerehabilitation, and could additionally include education, self-management support, nutritional counseling, or psychological support. A simple recommendation to increase physical activity, without a structured and reproducible training component, was not considered sufficient for inclusion.

Comparator. Eligible comparators were usual care, no structured rehabilitation, waiting list control, or minimal education without a structured exercise-based rehabilitation program.

Study design and report eligibility. Individually randomized parallel-group trials and cluster-randomized trials were eligible. Cluster-randomized trials were required to report or permit an analysis accounting for clustering. Quasi-randomized studies were excluded. Crossover trials were excluded because clinically meaningful carry-over effects of rehabilitation could persist beyond the first treatment period. Single-group pre–post studies, non-randomized controlled studies, observational studies, case reports, case series, reviews, and protocols were excluded. Conference abstracts, posters, and trial register records could be used to identify potentially eligible studies but were not considered outcome reports unless sufficient results were available for eligibility assessment and effect estimation. No publication date restriction was applied. Eligibility was restricted to reports published in English because translation resources were unavailable; this restriction is acknowledged as a potential source of language bias. Lack of immediate full-text access was not used as an exclusion criterion; records were actively reconciled against the screening log and assessed using the fullest available report or registry information.

### 2.3. Information Sources and Search Strategy

Electronic searches were conducted from database inception to 13 July 2026 in PubMed/MEDLINE, Scopus, Web of Science Core Collection, the Cochrane Central Register of Controlled Trials (CENTRAL), and the Physiotherapy Evidence Database (PEDro). A supplementary update through 28 August 2026 used the same revised population, rehabilitation, and randomized trial concepts: PubMed was rerun directly, CENTRAL and PEDro were rechecked using their public interfaces, and recent candidate publications were cross-verified against Scopus and Web of Science records. On 5 September 2026, a final targeted bibliographic update covering recent Scopus- and Web of Science-indexed literature was performed using the prespecified search concepts and publication-level verification by title and DOI; this identified the newly published Sharma et al. randomized trial [9]. ClinicalTrials.gov and the WHO International Clinical Trials Registry Platform (ICTRP) were last searched on 28 August 2026 and linked to publications by registration number, title, investigators, setting, and intervention. Across the supplementary updates, three additional eligible published RCT reports were identified: Akylbekov et al. (2026) [10], Muthukrishnan and Rajadurai (2026) [11], and Sharma et al. (2026) [9]. Because the reconciled original screening log was report-based and exact native update-export yields from the institutional Scopus and Web of Science interfaces were not retained in the working search record, update records were documented as an additional report pathway in the PRISMA diagram rather than merged numerically with the original database counts. Reference lists of included reports and relevant reviews were also screened. Complete source-specific search strings are provided in Supplementary Table S2. The information sources and final search coverage are summarized in Table 1.

During revision, the search syntax was audited for reproducibility and date-valid controlled vocabulary. Because “Pulmonary Rehabilitation” was not a MeSH descriptor in 2026, it has been removed as a controlled vocabulary term from the reported PubMed/MEDLINE and CENTRAL strategies. In the revised Supplementary Table S2, “pulmonary rehabilitation” and “respiratory rehabilitation” are retained as free-text concepts, together with controlled vocabulary headings that were valid at the time of the 2026 searches (including Respiratory Therapy, Breathing Exercises, and Exercise Therapy). A complete rerun of all bibliographic sources was not feasible during the present revision, particularly for subscription-based Scopus and Web of Science. We therefore did not retrospectively estimate or reconstruct source-specific retrieval counts that were not preserved in the original working search record.

### 2.4. Study Screening and Eligibility Assessment

Search outputs were consolidated in a review database, and duplicate records were removed before screening. Two reviewers (M.V.P. and M.I.) independently screened titles and abstracts and subsequently assessed the full text or the most complete available report of potentially eligible studies. Disagreements were resolved through discussion and consensus; no third-reviewer adjudication was required. The reconciled original screening log contained 76 reports assessed for eligibility. Supplementary updates were handled as a separate report-level pathway: candidate publications and trial registrations were linked to the original report set using title, DOI, authorship, setting, recruitment dates, and trial identifiers. Three additional eligible published RCT reports were identified and integrated into the qualitative synthesis: Akylbekov et al. (2026) [10], Muthukrishnan and Rajadurai (2026) [11], and Sharma et al. (2026) [9]. The final update also rechecked other newly published candidates; a single-session diaphragmatic-stretching RCT remained excluded because it did not meet the prespecified definition of a structured rehabilitation program. Multiple reports from the same underlying study were linked rather than counted as independent trials; because independence of the two Orooj populations [12,13] could not be verified despite attempted author contact, Orooj et al. (2025) [13] was excluded from the conservative primary synthesis and retained only as sensitivity evidence. The reconciled report-level eligibility dispositions are provided in Supplementary Table S3, and the dispositions of potentially relevant trial registrations are presented in Supplementary Table S4. Because Sharma et al. (2026) [9] was identified only in the final bibliographic update, its eligibility was rechecked explicitly against each prespecified PICOS element before inclusion; the criterion-by-criterion verification is provided in Supplementary Table S6.

### 2.5. Outcomes

The main outcome included the exercise capacity assessed directly after the completion of the rehabilitation program. Validated field exercise tests were eligible for inclusion, including the six-minute walk test (6MWT) and the Incremental Shuttle Walk Test (ISWT). When more than one eligible exercise capacity measure was reported at the same time point, the trial-prespecified primary exercise capacity measure was prioritized, and if no hierarchy was specified by the trial, the 6MWT was preferred to the ISWT and then another validated field test.

Secondary outcomes specified in the public record were pulmonary function (including FEV_1_, FVC and FEV_1_/FVC), dyspnea, health-related quality of life, inspiratory muscle strength assessed by maximal inspiratory pressure (MIP), adverse events, hospitalizations, and respiratory exacerbations. Peripheral muscle strength, when reported by individual trials, was extracted as an additional functional outcome and was not treated as a prespecified secondary outcome. The principal time point for quantitative synthesis was the end of the intervention. Longer-term follow-up outcomes were extracted and summarized separately and were not combined with immediate post-intervention data. For interpretation, higher values indicated benefit for exercise capacity, pulmonary function, muscle strength, and positively scored quality-of-life scales, whereas lower values indicated benefit for dyspnea and instruments such as the St George’s Respiratory Questionnaire (SGRQ). Effect directions were harmonized before pooling.

### 2.6. Data Extraction and Management

Following full-text assessment, data were extracted independently by two reviewers (M.V.P. and M.I.) into a structured extraction matrix. Disagreements were resolved through discussion and consensus; no third-reviewer adjudication was required. Extracted variables included study authors and year, country and setting, study design, participant characteristics, sample size, criteria used to define PTLD, intervention and comparator characteristics, program components, duration, frequency, intensity and supervision, outcome definitions and instruments, follow-up time points, numerical data required for effect estimation, attrition, and adverse events.

End-of-intervention data were prioritized for the primary analyses, whereas follow-up data were recorded separately. Potentially duplicate publications were evaluated using the linkage criteria described above. Sharma et al. (2026) [9] contained two eligible device-assisted intervention arms (Lung Flute and Acapella) sharing one control group. Because its exercise capacity, dyspnea, pulmonary function, and quality-of-life results were synthesized narratively and were not entered as separate pairwise estimates in any pooled model, the shared control group was not double-counted, and no control group splitting was required. All other included trials contained no more than two eligible randomized arms. When a standard error or confidence interval was reported instead of a standard deviation, the standard deviation could be derived using standard algebraic relationships when the required information was available. Missing standard deviations were not otherwise imputed. Medians were not converted to means unless sufficient distributional information was available, and no numerical values used in the meta-analyses were digitized from figures. When data were unclear, internally inconsistent, or unavailable, they were recorded as not reported or excluded from the relevant quantitative synthesis. Study authors were not contacted for clarification during the original review. During manuscript revision, the corresponding author of the two Orooj reports [12,13] was contacted specifically to clarify potential participant overlap and internally inconsistent numerical data; no response had been received by the time of revision. In response to source-level numerical inconsistencies identified during revision, Orooj et al. (2023) [12] and Sharma et al. (2026) [9] were additionally examined using a structured trustworthiness assessment informed by INSPECT-SR [14]. The assessment focused on internal consistency of reported baseline and outcome data, plausibility of statistical results, and whether the published analyses supported the stated comparative interpretation. No source values were corrected or reconstructed. Unresolved concerns were handled through sensitivity analyses and more cautious narrative interpretation; the structured assessment is reported in Supplementary Table S8.

### 2.7. Risk-of-Bias Assessment

Risk of bias was assessed using the Cochrane Risk of Bias 2 (RoB 2) tool for the effect of assignment to intervention [15]. Assessments were outcome-specific. Exercise capacity RoB 2 judgments were completed for all seven eligible randomized trial reports. Outcome-specific RoB 2 judgments were additionally completed for the principal secondary outcomes contributing to the Summary of Findings: dyspnea, health-related quality of life, pulmonary function, inspiratory muscle strength (MIP), and adverse events. Peripheral muscle strength was retained as an additional extracted functional outcome rather than a prespecified Summary of Findings outcome. The five domains were the randomization process, deviations from intended interventions, missing outcome data, measurement of the outcome, and selection of the reported result. Domain-level and overall judgments were categorized as low risk, some concerns, or high risk. For participant-reported outcomes, the participant was treated as the outcome assessor; therefore, lack of participant blinding was considered specifically within the outcome measurement domain, even when follow-up examiners were blinded. Assessments were performed independently by M.V.P. and M.I.; disagreements were resolved by consensus, and no third-reviewer adjudication was required. Traffic light presentation followed robvis conventions [16]. Detailed secondary outcome judgments are provided in Supplementary Table S5.

### 2.8. Data Synthesis and Statistical Analysis

The quantitative syntheses were conducted in Python 3.13.5 using NumPy 2.3.5, SciPy 1.17.0, and pandas 2.2.3 with a reproducible custom script implementing the procedures described below. All tests were two-sided, and *p* < 0.05 was considered statistically significant. Quantitative synthesis was undertaken when at least two studies were clinically and methodologically comparable for a given outcome. As an additional exploratory analysis prompted by identification of extractable MIP data in Torres et al. (2025) [17], unadjusted between-group differences in MIP change were synthesized for Torres et al. (2025) [17] and Muthukrishnan and Rajadurai (2026) [11]. For Torres, within-arm change standard errors were reconstructed from published 95% confidence intervals using group-specific t quantiles; the independent-arm change variances were summed. For Muthukrishnan [11], change-score standard errors were based on reported change standard deviations and randomized group sizes (60/60). A REML random-effects model with a Hartung–Knapp interval (df = 1) was used. The discrepancy between reconstructed Torres precision and its published between-group *p*-value is explicitly acknowledged; this analysis was not prespecified and is exploratory.

For continuous outcomes measured on the same scale and reported using comparable adjusted between-group estimands, mean differences (MDs) with 95% confidence intervals (CIs) were prioritized. After the final update, two independent trials reported adjusted between-group changes in ISWT distance; these estimates were therefore used for the primary scale-specific meta-analysis. Standard errors were derived from the reported 95% CIs. When conceptually equivalent outcomes were measured using different instruments but only post-intervention group means and standard deviations were available, standardized mean differences with Hedges’ small-sample correction (Hedges’ g) were calculated [16]. A cross-instrument end-of-intervention SMD analysis was used only as an exploratory sensitivity analysis because it mixes ISWT and 6MWT and does not preserve trial-level covariate adjustment. Positive values favor rehabilitation for exercise capacity. The adjusted ISWT model was not specified as the primary quantitative model in the registered protocol/public record. This analytical choice was made after the final evidence search identified two independent RCTs reporting directly comparable adjusted between-group ISWT change estimates (Katagira et al. (2026) [18] and Akylbekov et al. (2026) [10]. The adjusted ISWT analysis was prioritized because it preserved a common outcome scale and trial-level covariate adjustment, whereas the exploratory cross-instrument endpoint SMD approach combined different exercise capacity instruments and unadjusted post-intervention values. Although a standard error can be derived from a reported confidence interval, an adjusted between-group 6MWD effect was not combined with unadjusted endpoint or differently defined change estimates solely on that basis; pooling was restricted when the underlying estimands were not sufficiently comparable.

Random-effects inverse variance models were used because clinical and program-level heterogeneity was expected. Between-study variance (τ^2^) was estimated by restricted maximum likelihood (REML). Hartung–Knapp adjustment was used for confidence intervals and two-sided tests, with a t distribution and k−1 degrees of freedom [19]. Statistical heterogeneity was summarized using Cochran’s Q, τ^2^, and I^2^. A prediction interval was not calculated for the updated primary ISWT synthesis because only two studies contributed directly comparable adjusted effects. For exploratory secondary meta-analyses containing only two reports, the modified Hartung–Knapp variance multiplier was constrained to at least 1 to avoid spuriously narrow confidence intervals [20].

Robustness was examined using exploratory cross-instrument sensitivity analyses based on end-of-intervention values and by varying the treatment of the two potentially overlapping Orooj reports [12,13]. The conservative evidence set excluded Orooj et al. (2025) [13] because participant independence could not be confirmed and the report was judged to be at high overall risk of bias. Secondary outcomes were pooled only when at least two independent reports provided sufficiently comparable data and estimands; otherwise, adjusted trial-level effects were summarized narratively. Formal tests for small-study effects were not performed because fewer than 10 reports were available. Binary safety outcomes were not pooled because adverse event definitions and ascertainment were insufficiently comparable. The protocol/public record also anticipated subgroup and sensitivity analyses when sufficient comparable studies were available. Prespecified subgroup analyses were not undertaken because the final evidence base did not contain enough clinically and methodologically comparable RCTs within any meaningful subgroup. Planned sensitivity analyses that required larger numbers of comparable trials were likewise not feasible; the sensitivity analyses that could be performed with the final evidence set instead addressed cross-instrument endpoint synthesis, possible participant overlap between the Orooj reports, and source-level trustworthiness concerns. These deviations and their reasons are summarized in Supplementary Table S9 (PRISMA 2020 Item 24c).

### 2.9. Certainty of Evidence

Certainty of evidence was evaluated using the GRADE approach [21,22]. Randomized evidence started at high certainty and was downgraded for risk of bias, inconsistency, indirectness, imprecision, and publication bias. For adverse events, differences in the ascertainment and reporting process were considered under risk of bias, whereas differences in the definition and scope of the safety outcome (e.g., any adverse events versus serious or intervention-related adverse events) were considered under indirectness. M.V.P. and M.I. independently completed formal GRADE assessments for the prespecified exercise capacity body of evidence and for the principal secondary outcomes after completion of outcome-specific RoB 2 judgments; disagreements were resolved through discussion and consensus. For exercise capacity, all independent eligible RCTs in the conservative evidence set that reported the outcome were considered in the GRADE body, whereas only the two trials with directly comparable adjusted ISWT effects contributed to the primary scale-specific meta-analysis. When a coherent pooled estimate was unavailable, certainty was judged for the body of randomized comparative evidence supporting the prespecified outcome, and the result was summarized narratively rather than generating a new pooled effect solely for GRADE. Hospitalizations and respiratory exacerbations were not graded because eligible trials did not provide sufficiently informative comparative results.

## 3. Results

### 3.1. Study Selection

**Figure 1:**
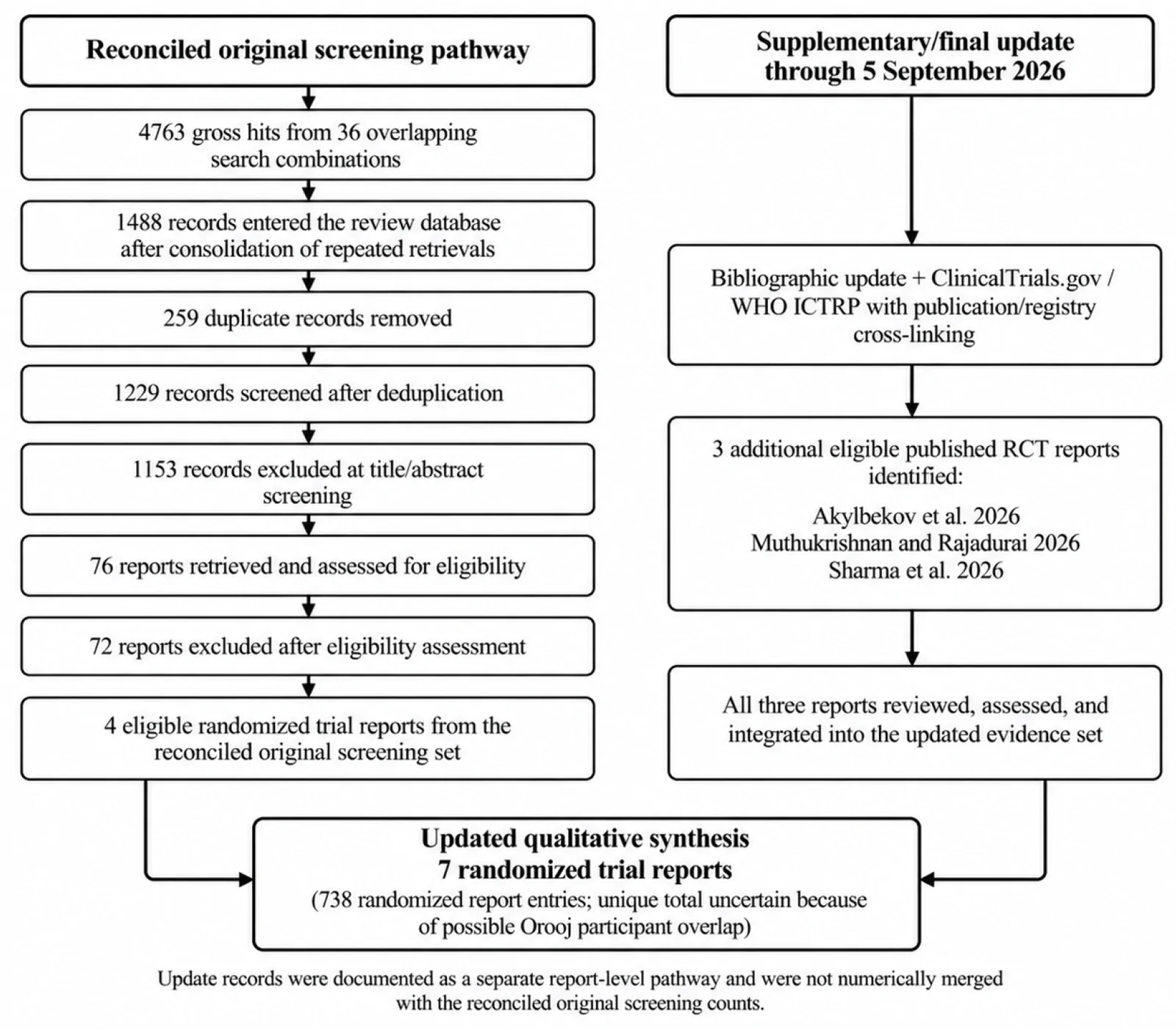
PRISMA 2020-style flow diagram of study selection and final bibliographic update. The 36 overlapping search combinations generated 4763 gross hits. After consolidation of repeated retrievals, 1488 records entered the review database; 259 duplicates were removed, leaving 1229 records for title/abstract screening. Of these, 1153 were excluded, and 76 reports were retrieved and assessed for eligibility; 72 were excluded, leaving four eligible randomized trial reports in the reconciled original screening set. Supplementary bibliographic and registry updating identified three additional eligible published randomized trial reports—Akylbekov et al. (2026) [10], Muthukrishnan and Rajadurai (2026) [11], and Sharma et al. (2026) [9]—yielding seven randomized trial reports in the updated qualitative synthesis. Update-search records are shown as a separate report-level pathway because exact native update-export yields were not merged with the reconciled original screening counts.

The 36 search combinations generated 4763 gross hits. Because these search combinations intentionally overlapped, this figure represents the summed outputs of multiple query formulations rather than 4763 unique bibliographic records. Consolidation of repeated retrievals across the search iterations yielded 1488 records in the review database. Formal cross-record deduplication subsequently removed 259 duplicates, leaving 1229 records for title/abstract screening. Source-specific native export counts from the original searches were not retained in a form that permits reliable retrospective reconstruction; accordingly, database-specific counts were not estimated after the fact. The updated study selection process is shown in Figure 1.

### 3.2. Characteristics of Included Trials

Seven randomized trial reports from Uganda, Brazil, India, and Kyrgyzstan evaluated structured pulmonary rehabilitation, telerehabilitation, inspiratory muscle training, or device-assisted respiratory rehabilitation in adults after completion of anti-tuberculosis treatment [9,10,11,12,13,17,18]. Reported randomized sample sizes ranged from 30 to 180 participants. Interventions lasted from 1 month to 12 weeks and included aerobic or walking exercise, resistance training, breathing or inspiratory muscle training, education/self-management, and, in the Sharma trial [9], repeated use of Lung Flute or Acapella airway-clearance devices with supplementary breathing and thoracic-expansion exercises. Exercise capacity was assessed using the ISWT in the Uganda and Kyrgyzstan trials, 6MWT in several reports, and Queens College Step Test-estimated VO_2_max in Sharma et al. (2026) [9]. The three reports integrated after the reconciled original screening set were Akylbekov et al. (2026) [10], a 114-participant single-blind RCT of 6-week culturally adapted pulmonary rehabilitation in Kyrgyzstan; Muthukrishnan and Rajadurai (2026) [11], a 120-participant trial of 12-week pulmonary rehabilitation plus threshold inspiratory muscle training in India; and Sharma et al. (2026) [9], a three-arm 180-participant RCT comparing Lung Flute, Acapella, and standard post-treatment care over 1 month in India.

An explicit PICOS recheck confirmed Sharma et al. (2026) [9] as eligible. Participants were AFB-negative, clinically stable adults who had completed anti-tuberculosis treatment and had ongoing dyspnea at baseline; the device arms constituted structured respiratory rehabilitation with supervised device training, adherence monitoring, and supplementary breathing/thoracic-expansion exercises; the comparator was standard post-treatment care without an airway-clearance device; the study used a three-arm parallel randomized design; and end-of-intervention exercise capacity and prespecified secondary outcomes were reported. Detailed verification is provided in Supplementary Table S6. Allocation concealment and blinding limitations were treated as risk-of-bias issues rather than eligibility failures.

The two reports by Orooj et al. [12,13] each randomized 90 participants and shared authorship and institutional features. Their participant populations could not be confirmed as independent, despite direct contact with the corresponding author and no response by the time of revision. To avoid potential double counting, the conservative evidence set retained Orooj et al. (2023) [12] and excluded Orooj et al. (2025) [13] from the primary quantitative synthesis; the latter was retained only for sensitivity and descriptive analyses. Key characteristics of the included randomized trial reports are summarized in Table 2, while selected comparative outcomes are presented in Table 3. The required interval between completion of anti-tuberculosis treatment and trial enrollment was inconsistently specified across studies. The Orooj reports required a minimum interval of approximately 6 months, whereas most other trials required completion of anti-tuberculosis treatment but did not define a specific minimum post-treatment interval [12,13].

### 3.3. Risk of Bias

**Figure 2:**
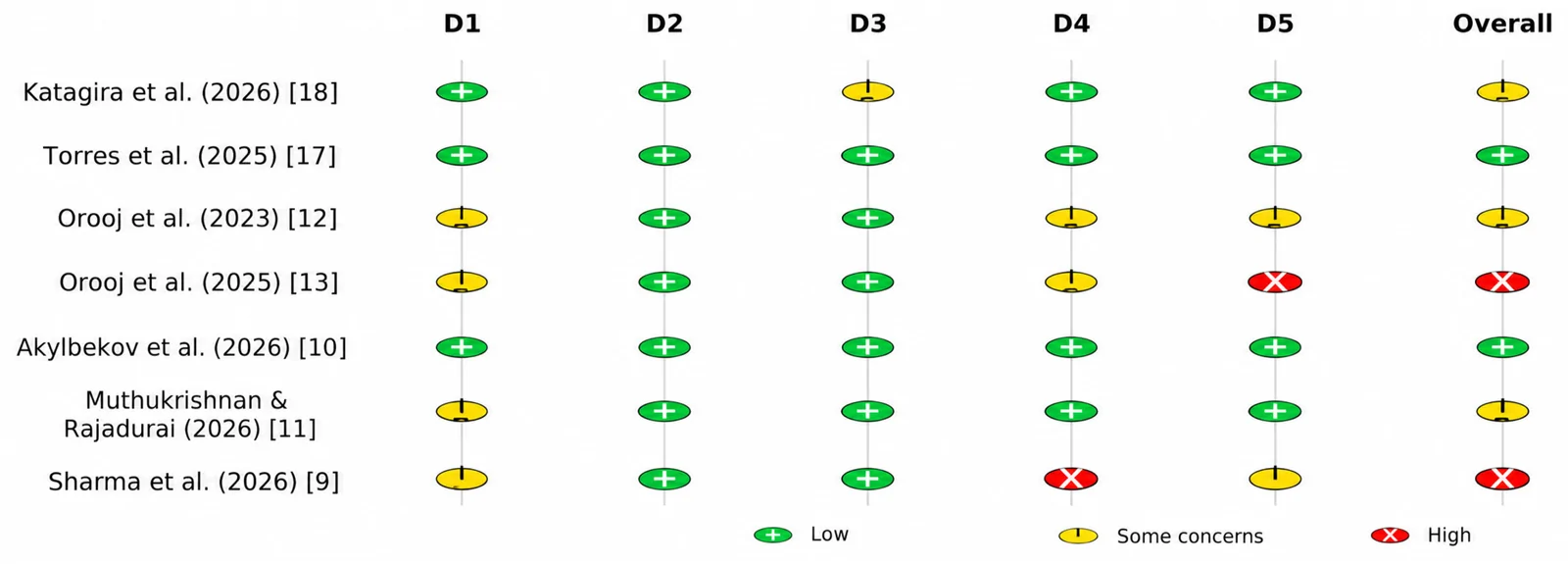
Traffic light plot of RoB 2 judgments for immediate post-intervention exercise capacity across the seven included randomized trial reports [9,10,11,12,13,17,18]. Green indicates low risk of bias, yellow indicates some concerns, and red indicates high risk of bias. D1: randomization process; D2: deviations from intended interventions; D3: missing outcome data; D4: measurement of the outcome; D5: selection of the reported result.

**Figure 3:**
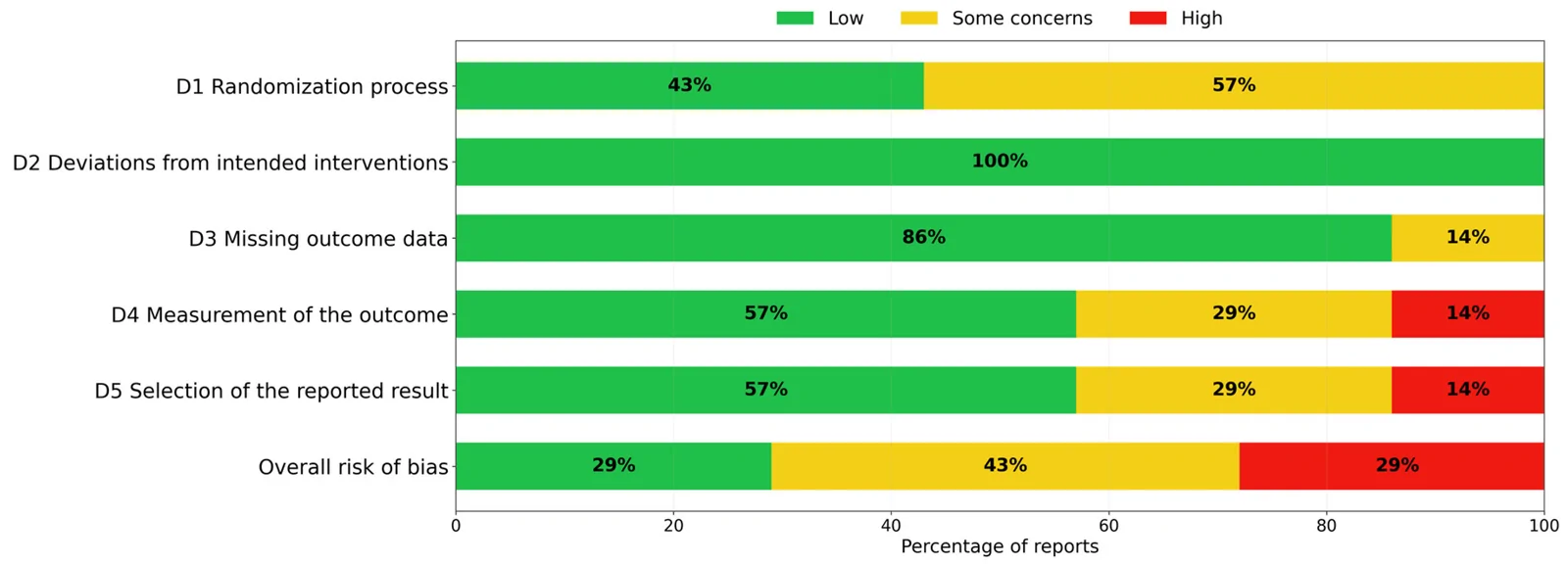
Unweighted summary of RoB 2 judgments across the seven included reports for immediate post-intervention exercise capacity. Percentages were rounded to the nearest whole number; therefore, the sum of displayed percentages may differ slightly from 100%.

Risk-of-bias judgments for immediate post-intervention exercise capacity were completed for all seven eligible randomized trial reports (Table 4; Figure 2). Torres et al. (2025) [17] and Akylbekov et al. (2026) [10] were judged to be at low overall risk of bias. Katagira et al. (2026) [18] raised some concerns, principally because 6-week ISWT observations were unavailable for 22 of 114 randomized participants despite an intention-to-treat mixed-model analysis. Orooj et al. (2023) [12] raised some concerns because of limited reporting of allocation procedures, assessor blinding, and prospective analysis planning. Orooj et al. (2025) [13] was judged to be at high overall risk of bias because of concerns about randomization reporting, outcome measurement, internal numerical inconsistencies, and selective reporting. Muthukrishnan and Rajadurai (2026) [11] raised some concerns because allocation concealment details were insufficiently reported. Sharma et al. (2026) [9] was judged at high overall risk for exercise capacity: allocation was based on participant-drawn chits without clearly documented concealment, neither participants nor assessors were blinded, the effort-dependent step test outcome could be influenced by knowledge of allocation, and no prospective registration or detailed analysis plan was identified in the report. The distribution of domain-level and overall RoB 2 judgments across the included reports is summarized in Figure 3.

Outcome-specific RoB 2 assessment changed the interpretation for several secondary results (Supplementary Table S5). For dyspnea and health-related quality of life, contributing participant-reported results generally raised concerns because participants knew their allocation; Sharma et al. (2026) [9] was judged at high risk for these outcomes because both participants and assessors were unblinded and the outcomes were susceptible to allocation awareness. Katagira et al. (2026) [18] also had incomplete 6-week outcome data. For pulmonary function, Torres et al. (2025) [17] was judged low risk, whereas Orooj et al. (2023) [12] and Muthukrishnan and Rajadurai (2026) [11] raised some concerns. Sharma PEFR was judged to raise some concerns because allocation concealment and prospective reporting information were limited, although the outcome was objectively recorded. The peripheral muscle strength result from Torres et al. (2025) [17] was judged low risk. Adverse event results raised some concerns because definitions and ascertainment were insufficiently standardized; Sharma [9] did not report a distinct adverse event outcome. Orooj et al. (2025) [13] was excluded from the conservative secondary outcome evidence set because participant independence from the 2023 report remained unresolved; Orooj et al. (2023) [12] did not explicitly report an adverse event outcome and therefore had no safety-result RoB 2 judgment.

### 3.4. Primary Outcome: Exercise Capacity

After the final update, two independent trials reported directly comparable adjusted between-group changes in ISWT distance and were therefore used for the primary scale-specific quantitative synthesis: Katagira et al. (2026) [18] and Akylbekov et al. (2026) [10]. Each trial randomized 114 adults. Katagira reported an adjusted between-group difference in ISWT change of +54.36 m (95% CI 17.22 to 91.51; *p* = 0.004), whereas Akylbekov [10] reported +123.0 m (95% CI 81.1 to 164.8; *p* < 0.001). Both effects favored rehabilitation, although their magnitudes differed substantially. Baseline demographic and clinical characteristics of the two trials contributing to the primary ISWT meta-analysis are compared in Supplementary Table S7.

The REML random-effects model pooling these adjusted ISWT effects yielded a mean difference of 87.98 m (95% Hartung–Knapp CI −348.01 to 523.96; *p* = 0.237). Between-study variance was substantial (τ^2^ = 1948.19 m^2^), and heterogeneity was high (Q = 5.78, df = 1, *p* = 0.016; I^2^ = 82.7%). The unusually wide Hartung–Knapp confidence interval was verified by recalculating the pooled estimate from the reported adjusted between-group effects and their derived standard errors. The recalculation reproduced the reported pooled estimate and confidence interval. This substantial imprecision reflects the combination of only two contributing trials, considerable between-study heterogeneity (I^2^ = 82.7%; τ^2^ = 1948.19 m^2^), and the use of a t distribution with only one degree of freedom in Hartung–Knapp inference. Consequently, the pooled estimate should not be interpreted as a precise estimate of the expected treatment effect. A prediction interval was not calculated with only two studies.

**Figure 4:**
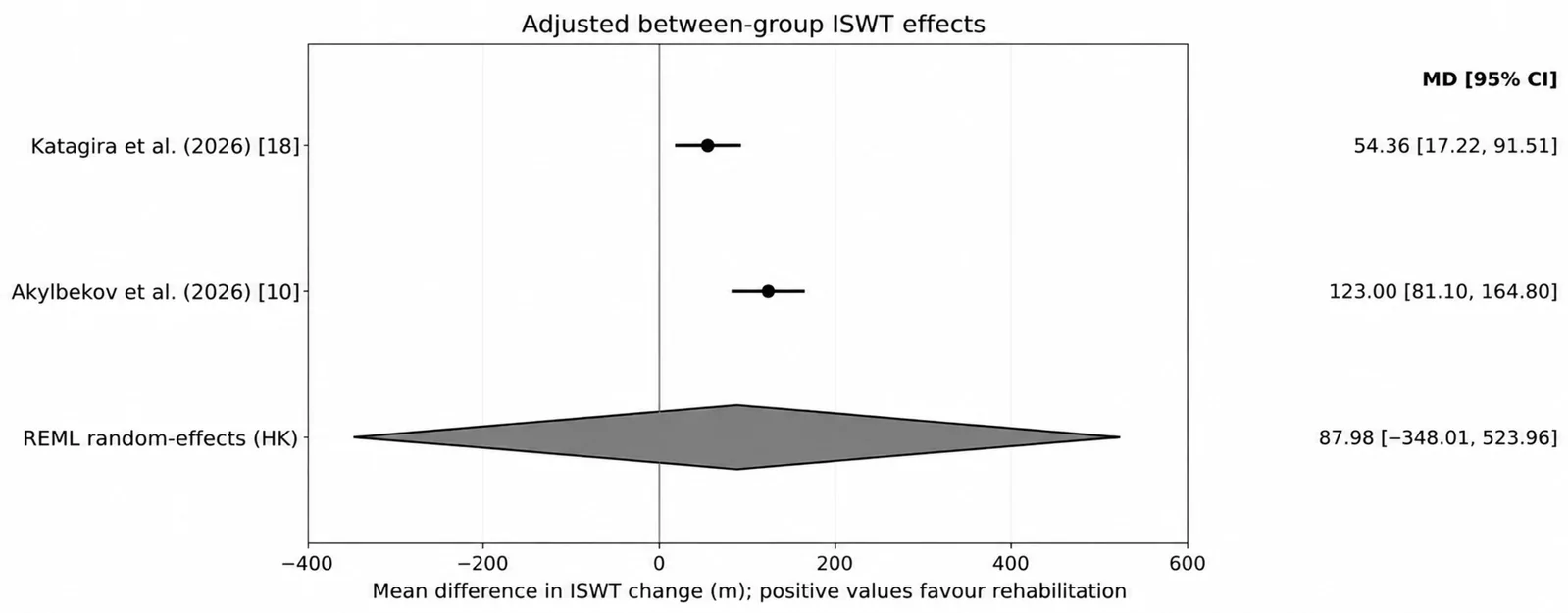
Forest plot of adjusted between-group ISWT change for the primary scale-specific meta-analysis [10,18]. Study estimates are adjusted mean differences with 95% confidence intervals; the pooled estimate uses a REML random-effects model with Hartung–Knapp inference. The very wide pooled interval reflects the small number of contributing trials and substantial heterogeneity.

Evidence from other exercise capacity instruments was directionally supportive but was not combined with the adjusted ISWT meta-analysis because the estimands and scales were not sufficiently comparable. Muthukrishnan and Rajadurai (2026) [11] reported an adjusted 12-week between-group 6MWD benefit of +87.4 m (95% CI 74.2 to 100.6; *p* < 0.001). Torres et al. (2025) [17] reported a favorable within-group 6MWD increase but a non-significant between-group comparison of change (*p* = 0.133), whereas Orooj et al. (2023) [12] reported a large between-group improvement (*p* = 0.001). Sharma et al. (2026) [9] reported a significant omnibus three-group difference in Queens College Step Test-estimated VO_2_max (F(2,177) = 69.25; *p* < 0.001; η^2^ = 0.44); however, the randomized arms were substantially imbalanced at baseline, the control group pre/post values were identical to two decimals in the published table, and the post-intervention Lung Flute mean was below the control mean. These data were therefore retained only as cautious narrative evidence and were not interpreted as demonstrating a uniform device-versus-control benefit. Orooj et al. (2025) [13] was not used in the primary evidence set because participant independence from the 2023 report could not be established. Individual study estimates and the pooled adjusted ISWT effect are presented in Table 5 and Figure 4. Although the standard error for the Muthukrishnan adjusted 6MWD estimate can be derived from its 95% confidence interval [11], we did not enter it into the exploratory 6MWD endpoint-value model because that would combine an adjusted between-group estimand with unadjusted endpoint/differently defined estimates from other trials.

### 3.5. Sensitivity Analyses

A cross-instrument synthesis based on end-of-intervention group values was performed as an exploratory sensitivity analysis rather than as the primary inferential model. In the conservative three-report scenario (Katagira (2026) [18], Torres (2025) [17], and Orooj (2023) [12], the pooled Hedges’ g was 1.38 (95% Hartung–Knapp CI −3.25 to 6.01; *p* = 0.329; I^2^ = 97.4%). When both Orooj reports were included, the pooled estimate was g = 1.94 (95% CI −1.08 to 4.97; *p* = 0.133; I^2^ = 97.4%); when Orooj (2023) [12] was replaced by Orooj (2025) [13], it was g = 1.42 (95% CI −3.40 to 6.24; *p* = 0.332; I^2^ = 97.5%). These scenarios remained highly heterogeneous and imprecise and are presented only to demonstrate the sensitivity of cross-instrument endpoint synthesis to study selection and possible participant overlap. The exploratory sensitivity analyses are summarized in Table 6. The 12-month outcomes reported by Orooj et al. (2025) [9] are provided in Supplementary Table S1. A further trustworthiness sensitivity analysis considered the evidence with Orooj et al. (2023) [12] and Sharma et al. (2026) [9] omitted. Neither report contributed to the primary adjusted ISWT meta-analysis, so the primary pooled estimate (+87.98 m; 95% Hartung–Knapp CI −348.01 to 523.96) was unchanged. Excluding Orooj et al. (2023) [12] from the exploratory 6MWD meta-analysis left only Torres et al. (2025) [17] and therefore precluded pooling; excluding Sharma et al. (2026) [9] did not alter any pooled estimate because that trial had been synthesized narratively. Thus, removal of the two reports does not change the primary meta-analytic result, but it further reduces the amount of supportive evidence available for broader exercise capacity and secondary outcome interpretations. No subgroup analysis was performed because too few sufficiently comparable trials were available within any prespecified clinical or intervention subgroup. The feasible sensitivity analyses and deviations from the registered analysis plan are summarized in Supplementary Table S9. A separate risk-of-bias-based sensitivity meta-analysis was not feasible for the primary adjusted ISWT synthesis because only two trials contributed directly comparable estimates. Akylbekov et al. (2026) [10] was judged at low overall risk of bias, whereas Katagira et al. (2026) [18] raised some concerns, primarily related to missing outcome data. Excluding the latter would leave only one trial, precluding quantitative pooling and preventing an assessment of the robustness of the pooled estimate through risk-of-bias-based study exclusion. Consequently, the potential impact of risk of bias on confidence in the findings was considered through outcome-specific RoB 2 assessments and explicitly incorporated into the GRADE certainty evaluation. The first two scenarios in Table 6 include Orooj et al. (2023) [12], and the alternative scenario includes Orooj et al. (2025) [13]; all are exploratory and depend on unresolved trustworthiness or participant overlap concerns.

### 3.6. Secondary Outcomes

Secondary outcome evidence was updated using the conservative independent report set. The pre-existing exploratory 6MWD meta-analysis of Torres (2025) [17] and Orooj (2023) [12] yielded a mean difference of 120.09 m (95% modified Hartung–Knapp CI −523.84 to 764.01; *p* = 0.254; I^2^ = 81.4%). Muthukrishnan and Rajadurai (2026) [11] was not added to the exploratory 6MWD endpoint-value model. Although its standard error can be derived from the published 95% confidence interval, the reported effect is an adjusted between-group estimand, whereas the available estimates contributing to the endpoint-value model are not directly equivalent. We therefore retained the adjusted 6MWD effect of +87.4 m (95% CI 74.2 to 100.6) as separate trial-level evidence rather than pooling non-equivalent estimands. Sharma et al. (2026) [9] used Queens College Step Test-estimated VO_2_max and was synthesized narratively rather than pooled with walking test outcomes. For dyspnea, the previous exploratory pooled standardized effect from Katagira (2026) [18] and Orooj (2023) [12] was Hedges’ g = −0.58 (95% modified Hartung–Knapp CI −6.11 to 4.95; *p* = 0.410; I^2^ = 87.6%). Akylbekov (2026) [10] additionally reported an adjusted MRC improvement of −1.1 points (95% CI −1.4 to −0.9); Muthukrishnan (2026) [11] reported favorable dyspnea outcomes; and Sharma (2026) [9] reported a significant three-group mMRC difference (F(2,177) = 59.23; *p* < 0.001), with the largest within-group reduction in the Lung Flute arm [9]. Orooj et al. (2023) [12] contributes to all four exploratory models in Table 7 (6MWD, dyspnea, SF-36 physical functioning and SF-36 mental health). Excluding it leaves a single report in each model and precludes pooling. These estimates are not confirmatory.

Two earlier independent reports (Torres (2025) [17] and Orooj (2023) [12] contributed SF-36 domain data. The exploratory pooled mean difference for physical functioning was 32.86 points (95% modified Hartung–Knapp CI −52.24 to 117.96; *p* = 0.128; I^2^ = 73.4%), and for mental health it was 22.63 points (95% CI 0.49 to 44.77; *p* = 0.049; I^2^ = 0%). These two-study analyses remain hypothesis-generating. Newer trials provide additional but differently measured quality-of-life evidence: Akylbekov (2026) [10] reported an adjusted EQ-5D-5L visual-analog-scale improvement of +20.2 points (95% CI 15.5 to 24.9), Muthukrishnan (2026) [11] reported an adjusted SGRQ total improvement of −14.8 points (95% CI −17.3 to −12.3), and Sharma (2026) [9] reported mixed SF-12 findings, with improvement in the Acapella arm but a decline in the Lung Flute arm. These results were not pooled because instruments, estimands, and multi-arm comparisons differed.

**Figure 5:**
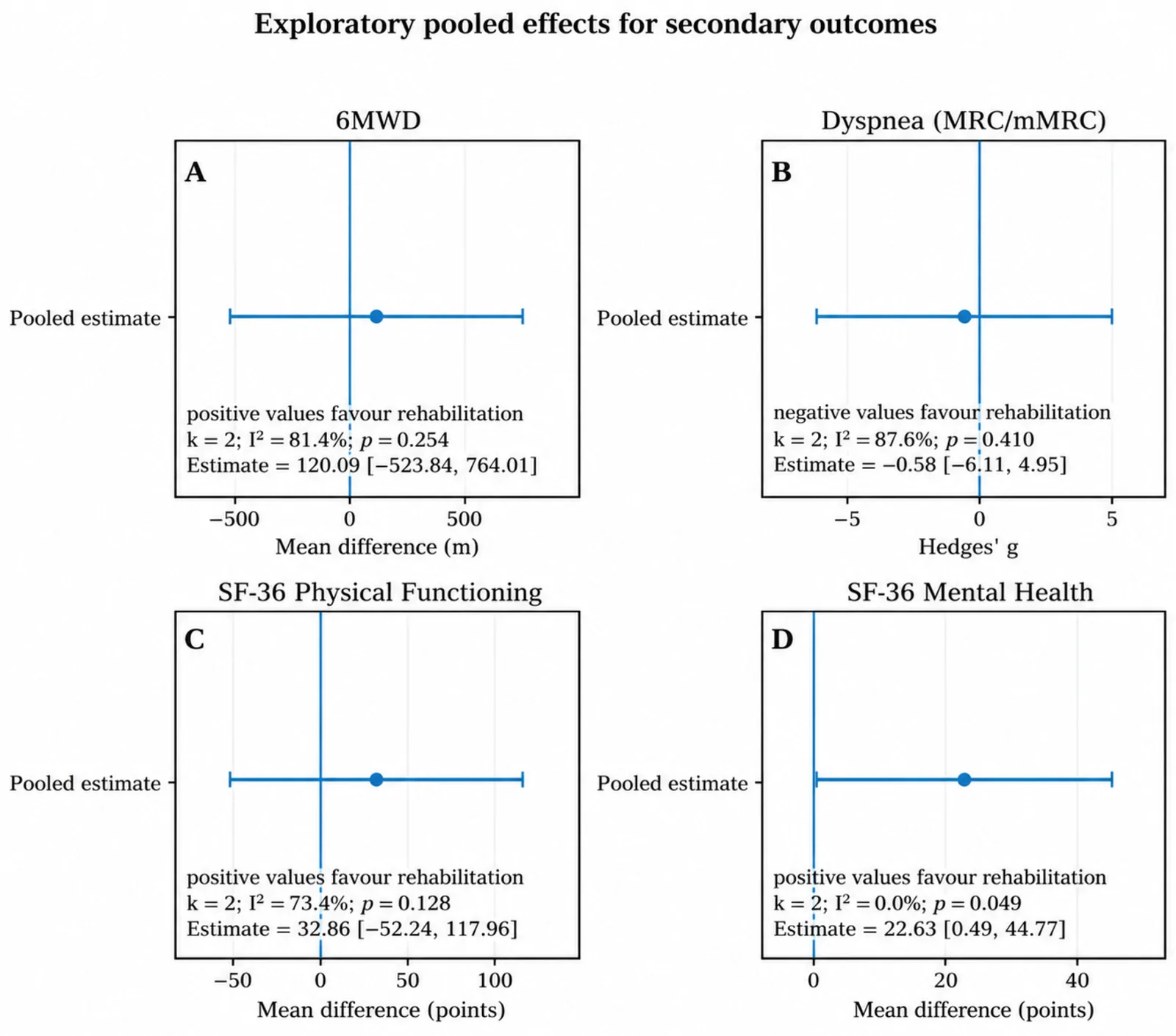
Summary plots of exploratory pooled effects for secondary outcomes using independent reports. Panel (**A**) 6MWD. Panel (**B**) dyspnea (MRC/mMRC). Panel (**C**) SF-36 physical functioning. Panel (**D**) SF-36 mental health. Points represent pooled estimates and horizontal bars represent modified Hartung–Knapp 95% confidence intervals. All four pooled estimates include Orooj et al. (2023) [12] and remain dependent on unresolved source-level trustworthiness concerns.

Pulmonary function outcomes were not pooled because measures and estimands were insufficiently comparable. Orooj (2023) [12] reported no significant independent post-intervention FEV_1_ difference (*p* = 0.142), whereas Muthukrishnan (2026) [11] reported adjusted between-group improvements of +0.21 L in FEV_1_ and +0.29 L in FVC. Sharma (2026) [9] reported a significant three-group PEFR difference (F(2,177) = 23.24; *p* < 0.001; η^2^ = 0.21), with the largest increase in the Acapella arm; PEFR was retained as a distinct pulmonary function measure and was not combined with spirometric volumes. Akylbekov (2026) [10] focused primarily on functional and patient-reported outcomes. Muscle function evidence was heterogeneous: Torres (2025) [17] reported favorable handgrip and knee-extension torque comparisons, while Muthukrishnan (2026) [11] reported an adjusted MIP benefit of +18.6 cmH_2_O (95% CI 15.1 to 22.1). Adverse event reporting remained inconsistent. Katagira reported 12/57 events in rehabilitation [18] versus 6/57 in usual care, including two serious events considered unrelated to rehabilitation; Torres reported no adverse events [17]; Orooj (2023) [12] did not explicitly report adverse event outcomes; Akylbekov [10] reported three adverse events in total (two intervention, one control), with only one episode of leg pain considered related to rehabilitation; Muthukrishnan [11] reported no serious adverse events; and Sharma [9] did not report a distinct adverse event outcome. These data do not support a pooled safety estimate. The exploratory pooled secondary outcomes are summarized in Table 7 and Figure 5, while outcomes not suitable for quantitative pooling are presented in Table 8. Inspiratory muscle strength was considered separately from peripheral muscle strength. Muthukrishnan and Rajadurai (2026)reported an adjusted between-group MIP improvement of +18.6 cmH_2_O (95% CI 15.1 to 22.1) [11]. The public trial record for Torres et al. specified assessment of maximal inspiratory and expiratory pressures, but the final publication did not provide a numerical MIP result suitable for comparative extraction; therefore, no Torres MIP estimate was synthesized. Handgrip strength and knee-extension torque from Torres were retained only as additional peripheral muscle outcomes [17]. Inspiratory muscle strength was reported by two trials (150 randomized participants). Torres et al. (2025) [17] reported MIP changes of +8.2 cmH_2_O (95% within-group CI 0.7 to 15.6) in the intervention arm (n = 14) and −3.9 cmH_2_O (95% within-group CI −13.6 to 5.7) in the control arm (n = 16), an unadjusted difference in mean changes of +12.1 cmH_2_O (published between-group *p* = 0.266). Muthukrishnan and Rajadurai (2026) [11] reported changes of +22.8 ± 7.4 versus +4.2 ± 3.8 cmH_2_O (n = 60/60), with an adjusted between-group difference of +18.6 cmH_2_O (95% CI 15.1 to 22.1). An exploratory change-score meta-analysis yielded MD +17.75 cmH_2_O (95% Hartung–Knapp CI −10.05 to 45.56; I^2^ approximately 20.6%). Reconstructed precision for Torres [17] could not be reconciled with its published *p*-value, so this pooled result remains exploratory. Peripheral handgrip and knee-extension strength are reported separately.

### 3.7. Certainty of Evidence and Summary of Findings

Certainty of evidence for exercise capacity was rated very low. GRADE considered six independent RCTs (648 randomized participants) in the conservative evidence set. Two RCTs (228 randomized participants) contributed directly comparable adjusted ISWT effects to the primary scale-specific meta-analysis, while four additional RCTs reported 6MWT or Queens College Step Test-estimated VO_2_max outcomes and were synthesized separately. Randomized evidence was downgraded one level for serious risk of bias because one independent RCT was at high risk and three raised some concerns, two levels for very serious inconsistency because study-level effect magnitudes, exercise capacity instruments, and estimands differed markedly and pooled analyses showed substantial unexplained heterogeneity, and one level for serious imprecision because the pooled ISWT confidence interval was extremely wide and crossed the null, although additional RCTs reported more precise favorable trial-level effects. Indirectness was not considered serious, and publication bias could not be formally assessed. Overall certainty therefore remained very low. In accordance with GRADE wording for very-low-certainty evidence, this body of evidence is therefore interpreted as very uncertain regarding the magnitude of the effect of structured rehabilitation on exercise capacity.

After completion of outcome-specific RoB 2 assessments, formal GRADE ratings were updated for the principal secondary outcomes (Table 9). Certainty remained very low for dyspnea: five independent RCTs reported the outcome, but the directly pooled two-report estimate was highly heterogeneous and extremely imprecise, and the newly added Sharma [9] result was at high outcome-specific risk of bias. Health-related quality-of-life evidence remained very low because six RCTs used different participant-reported instruments, Sharma [9] showed divergent findings across its two intervention arms, and several results had outcome measurement concerns. Pulmonary function evidence remained very low because four RCTs used non-equivalent measures/estimands, including PEFR in Sharma, and a coherent pooled estimate was unavailable. Inspiratory muscle strength (MIP) was graded separately and was rated very low certainty because only one included RCT provided a directly extractable comparative MIP estimate, that trial raised some risk-of-bias concerns, and the estimate was based on a single study. Peripheral muscle strength was not graded as a prespecified outcome and is reported only as an additional extracted functional outcome. Adverse event evidence remained very low. Risk of bias was considered serious because adverse event ascertainment and reporting were not standardized across trials. Indirectness was considered serious because the reported safety outcomes did not correspond to a uniform adverse event construct: some trials reported overall adverse events, whereas others reported only serious or intervention-related events, and event definitions and attribution to rehabilitation were incompletely specified. Imprecision was considered very serious because adverse events were sparse and no reliable comparative safety effect could be estimated. Publication bias could not be assessed. Hospitalizations and respiratory exacerbations were not graded because sufficiently informative comparative data were not reported. MIP was assessed using two RCTs (150 randomized). The exploratory pooled unadjusted mean change difference was +17.75 cmH_2_O (95% Hartung–Knapp CI −10.05 to 45.56; I^2^ approximately 20.6%). The inconsistency estimate is unreliable with only two studies. Very low certainty is retained provisionally because of imprecision, differences in intervention/estimands, outcome-specific bias concerns, and the mismatch between the reported and reconstructed Torres precision; a final judgment requires documented outcome-specific RoB 2 reassessment.

## 4. Discussion

### 4.1. Principal Findings

This systematic review identified seven randomized trial reports evaluating structured rehabilitation in adults after pulmonary tuberculosis [9,13,17,18]. Four reports were present in the reconciled 76-report screening set; the supplementary update through 28 August 2026 identified Akylbekov et al. (2026) [10] and Muthukrishnan and Rajadurai (2026) [11], and the final targeted bibliographic update through 5 September 2026 identified Sharma et al. (2026) [9]. Across the seven reports, there were 738 randomized report entries, although the number of unique participants remains uncertain because possible overlap between the two Orooj populations could not be resolved. In the conservative evidence set excluding Orooj (2025) [13], six reports randomized 648 participants. Trial-level exercise capacity findings were predominantly favorable but used ISWT, 6MWT, or step-test-derived VO_2_max. The two directly comparable adjusted ISWT trials are more informative when considered side by side: Katagira et al. reported +54.36 m (95% CI 17.22 to 91.51) [18], and Akylbekov et al. reported +123.0 m (95% CI 81.1 to 164.8). The pooled estimate (+87.98 m) is retained as a secondary synthesis, but its Hartung–Knapp confidence interval was extremely wide and included the null [10]. Muthukrishnan reported a favorable adjusted 6MWD effect [11]. Sharma [9] reported omnibus differences in step-test-estimated VO_2_max, but substantial baseline imbalance and unresolved control group reporting concerns prevent reliable causal interpretation. The findings do not establish a generalizable magnitude of benefit.

Secondary outcomes also showed a broader but heterogeneous pattern. Akylbekov reported improvements in MRC dyspnea, CAT, CCQ, and EQ-5D-5L visual-analog scores [10], while Muthukrishnan reported improvements in maximal inspiratory pressure, SGRQ total score, spirometry, and dyspnea [11]. Sharma reported significant omnibus between-group differences in mMRC and PEFR and divergent SF-12 changes across device arms; however, baseline imbalance, identical reported control group pre/post values, and uncertainty regarding device-versus-control contrasts limit interpretation [9]. Torres reported favorable SF-36 and muscle strength outcomes [17], and Orooj (2023) reported large improvements in 6MWD and dyspnea [12]. Outcome instruments, estimands, intervention formats, and reporting quality therefore differed substantially, preventing a single coherent quantitative synthesis across most secondary domains. Safety data remained too sparse and inconsistently ascertained for a robust pooled conclusion.

### 4.2. Interpretation and Sources of Heterogeneity

The substantial heterogeneity is clinically plausible. Participants differed in baseline functional limitation and in the type and severity of post-tuberculosis pulmonary sequelae, while interventions ranged from conventional multi-component pulmonary rehabilitation and telerehabilitation to inspiratory muscle training and device-assisted airway-clearance/respiratory exercise. Program duration ranged from 1 month to 12 weeks, and comparator intensity varied. Exercise capacity was measured using ISWT, 6MWT, or Queens College Step Test-estimated VO_2_max, and analyses differed between adjusted change estimates, endpoint comparisons, and multi-arm ANOVA. Even within the two comparable adjusted ISWT trials, effects differed from +54.36 m in Uganda to +123.0 m in Kyrgyzstan (I^2^ = 82.7%). The pooled estimate should therefore be interpreted as an average across two contextually distinct programs rather than a universal PTLD treatment effect. Accordingly, the magnitude of the pooled effect should not be extrapolated to other PTLD populations, rehabilitation programs, or healthcare settings without considerable caution, particularly given the substantial between-study heterogeneity (I^2^ = 82.7%) and the uncertainty associated with an estimate derived from only two trials.

Particular caution remains necessary for the two Orooj reports [12,13]. Both yielded unusually large effects, shared authorship and institutional characteristics, and could not be confirmed as independent participant populations. The corresponding author was contacted for clarification, but no response was received. The 2025 report also had high overall risk of bias and internal numerical inconsistencies. To minimize double counting, it was excluded from the conservative primary evidence set and retained only as sensitivity information. This decision prevents the main synthesis from assigning disproportionate influence to a potentially duplicated cohort.

A structured source-level trustworthiness assessment informed by INSPECT-SR identified unresolved concerns in Orooj et al. (2023) [12] and Sharma et al. (2026) [9] (Supplementary Table S8). For Orooj (2023) [12], the control group baseline 6MWD differs between published tables (352.37 versus 313.0 m), the reported baseline between-group *p*-value is difficult to reconcile with the summary statistics, and FEV_1_/FVC values around/above 100 have unclear scaling or units. For Sharma (2026) [9], all four reported control group outcomes are unchanged pre/post to two decimal places, baseline VO_2_max differs substantially across randomized arms, and the omnibus ANOVA does not by itself establish benefit of either device or control. These concerns relate to the source reports rather than the conduct of the present review and do not establish research misconduct; nevertheless, they reduce confidence in those trial-level findings and justify conservative interpretation and sensitivity analyses [14].

The final analytical approach illustrates why estimand choice matters. The exploratory cross-instrument meta-analysis standardized post-intervention values because change-score standard deviations were unavailable; however, endpoint values can underrepresent comparative improvement when baseline imbalance exists and do not preserve trial-level covariate adjustment. The availability of two adjusted ISWT effects allowed the primary synthesis to prioritize comparable between-group change estimates. This is methodologically preferable, but the small number of comparable trials produces very wide Hartung–Knapp uncertainty. Accordingly, the cross-instrument SMD model is presented only as a sensitivity analysis rather than as the main evidence estimate.

The relationship between functional outcomes and pulmonary function measures remains complex. PTLD can leave fixed obstructive, restrictive, mixed, or structural abnormalities that may not reverse substantially with rehabilitation [2,3]. Pulmonary rehabilitation instead targets modifiable consequences such as physical deconditioning, peripheral and respiratory muscle dysfunction, inefficient exercise responses, dyspnea-related activity avoidance, and self-management limitations [2,23,24]. The Muthukrishnan trial reported modest spirometric gains alongside larger functional, inspiratory muscle, and quality-of-life effects [11], whereas Sharma reported changes in PEFR, dyspnea, and exercise-related outcomes, but source-level inconsistencies and baseline imbalance limit device-versus-control interpretation [9]. Thus, clinically meaningful functional improvement in PTLD need not depend on major reversal of residual structural lung disease, and different rehabilitation components may influence different physiological domains.

Both adjusted ISWT trials reported effect estimates exceeding commonly cited ISWT thresholds derived from chronic respiratory disease populations. Katagira reported +54.36 m [18], and Akylbekov +123.0 m [10]. The 47.5-m threshold reported by Singh et al. for COPD pulmonary rehabilitation [25] provides useful context, while Akylbekov [10] used a 35-m ISWT MCID in its trial interpretation. However, a PTLD-specific ISWT MCID has not been firmly established. These comparisons should therefore be considered supportive clinical context rather than definitive proof that a universal PTLD threshold has been exceeded.

### 4.3. Comparison with Previous Evidence

The findings remain more conservative than broader syntheses that combine randomized and non-randomized designs. Gebremedhn et al. reported improvements in exercise capacity, pulmonary function, and health-related quality of life across heterogeneous study designs [4], and the 2026 review by Kumar et al. similarly found generally favorable rehabilitation outcomes [5]. The present review restricts eligibility to randomized controlled trials and prioritizes comparative between-group effects. Three reports identified after reconciliation of the original screening set expanded the direct randomized evidence, but the formal primary synthesis still contains only two trials with directly comparable adjusted ISWT estimands, which limits precision. Recent PTLD-specific studies provide additional clinical context for these findings. Silva et al. reported improvements in exercise capacity and pulmonary function following a multidisciplinary pulmonary rehabilitation program in a prospective multicenter study that included a non-rehabilitated comparison group [26]. Similarly, Rifat et al. reported favorable changes in exercise capacity and spirometric measures following a population-based rehabilitation program in Bangladesh [27]. Stenberg et al. also described improvements in functional capacity and respiratory symptoms following a community-based rehabilitation intervention in Malawi [28]. These findings support the feasibility and potential clinical value of pulmonary rehabilitation across different healthcare settings. However, differences in study design, comparator availability, and outcome assessment limit direct comparisons with the randomized evidence synthesized in the present review. As also highlighted by Kirakosyan et al., further rigorous research is needed to establish the effectiveness, optimal delivery, and broader implementation of rehabilitation for individuals with PTLD [29].

Current clinical standards for PTLD recognize the need to assess persistent respiratory and functional impairment and include rehabilitation among management options for symptomatic survivors [3]. Established pulmonary rehabilitation evidence from COPD and other chronic respiratory diseases also supports the biological plausibility of exercise-based rehabilitation and alternative delivery models, including telerehabilitation [24,25,30]. However, extrapolation should remain cautious. The PTLD-specific randomized evidence now includes supervised center-based programs, culturally adapted rehabilitation, home-based telerehabilitation, inspiratory muscle training, and device-assisted respiratory rehabilitation [9,10,11,12,13,17,18]. Direct head-to-head evidence comparing complete delivery models remains limited; the Sharma [9] trial directly compared two airway-clearance devices but did not compare comprehensive rehabilitation settings.

This distinction is especially relevant in high-TB-burden settings where specialized rehabilitation services may be limited. The Kyrgyzstan trial demonstrated that a culturally adapted 6- program can produce substantial functional and quality-of-life gains and reported favorable cost-effectiveness [10]. Home-based or remotely supported approaches may reduce access barriers, but the PTLD telerehabilitation evidence remains comparatively small. Future implementation work should therefore not only evaluate efficacy, but also adherence, scalability, equity, workforce requirements, and cost-effectiveness across delivery models.

### 4.4. Clinical Implications

Interpretation of Sharma et al. (2026) [9] should remain cautious because its omnibus results do not resolve important baseline and control group reporting concerns; estimates including Orooj et al. (2023) [12] are exploratory and hypothesis-generating.

The updated evidence supports considering structured rehabilitation for adults with persistent dyspnea, reduced exercise capacity, or functional limitation after completion of pulmonary tuberculosis treatment, particularly when impairment is identified through structured PTLD assessment [2]. Trial-level effects across conventional, remote, inspiratory muscle, and device-assisted approaches are often favorable, but certainty remains very low for exercise capacity and several patient-important outcomes. The newly identified Sharma trial broadens the intervention spectrum, but its device-specific effects should be interpreted cautiously because of marked baseline imbalance, unusual invariance of control group values, and uncertainty regarding the device-versus-control contrasts [9]. Current evidence does not establish a single optimal duration, exercise dose, component mix, device, supervision model, or delivery setting.

From a clinical implementation perspective, pulmonary rehabilitation should be considered within a broader post-tuberculosis care pathway rather than as an isolated intervention. Pontali et al. emphasized the importance of functional assessment and rehabilitation in addressing persistent respiratory and physical limitations after tuberculosis [31]. Similarly, Silva et al. highlighted the need to recognize PTLD as an important priority beyond microbiological cure [32]. These perspectives support the integration of symptom assessment, functional evaluation, and individualized rehabilitation planning into post-treatment follow-up. Nevertheless, given the very low certainty of the available randomized evidence, decisions regarding rehabilitation content, intensity, supervision, and delivery setting should remain individualized rather than based on assumptions of superiority among specific approaches.

Safety findings are encouraging but not definitive. No serious adverse events were reported in the Muthukrishnan trial [11]; Akylbekov reported only three adverse events overall, with one episode of leg pain considered related to rehabilitation [10]; and serious events in Katagira were judged unrelated to the rehabilitation intervention [18]. Sharma described discomfort management during supervised device use but did not report a distinct adverse event outcome [9]. Adverse event definitions and ascertainment therefore remain inconsistent across trials. Rehabilitation should be individualized with attention to residual lung impairment, comorbidities, exercise tolerance, oxygenation when clinically indicated, device tolerance where applicable, and standard pulmonary rehabilitation safety principles.

### 4.5. Strengths and Limitations

This review has several strengths. The protocol was prospectively registered in PROSPERO, reporting was aligned with PRISMA 2020 and PRISMA-S principles, eligibility was restricted to randomized controlled trials, and the previously inconsistent full-text log was reconciled to 76 traceable reports. Bibliographic and registry updating was extended through 28 August 2026, followed by a final targeted update of recent Scopus- and Web of Science-indexed literature through 5 September 2026, which identified a third additional eligible randomized report (Sharma et al. (2026) [9]). Exercise capacity RoB 2 was updated for all seven reports, and outcome-specific RoB 2 assessments were updated for the principal secondary outcomes included in the Summary of Findings. The primary quantitative synthesis prioritizes adjusted between-group effects on a common ISWT scale, the multi-arm Sharma [9] trial was handled without double counting its shared control, and the unresolved Orooj overlap was handled conservatively.

Important limitations remain. Only seven eligible randomized trial reports were identified, and the conservative evidence set contains six reports after excluding Orooj (2025) [13] because participant independence could not be verified. The formal primary meta-analysis contains only two directly comparable adjusted ISWT trials, so Hartung–Knapp inference is necessarily unstable and imprecise. Clinical and methodological heterogeneity remains substantial and is further increased by the inclusion of a three-arm device-assisted trial using step-test-estimated VO_2_max rather than a walking test. Eligibility was restricted to English-language reports, and Embase and CINAHL were not searched because institutional access was unavailable. The final update identified Sharma et al. by publication-level title/DOI verification in literature covered by Scopus and Web of Science; however, exact native update-export yields from authenticated institutional Scopus and Web of Science interfaces were not retained in the working search log. The update is therefore documented as a separate report-level pathway rather than being numerically merged with the original database counts.

An additional limitation concerns reproducibility of the original database-level record counts. Although the review database and screening totals were retained, source-specific native export counts from the original PubMed/MEDLINE, Scopus, Web of Science, CENTRAL, and PEDro searches were not preserved in a form that allows reliable retrospective reconstruction. We therefore report this limitation explicitly and do not estimate database-specific counts retrospectively.

Outcome reporting also limited synthesis. The Muthukrishnan trial reported adjusted 6MWD, MIP, SGRQ, FEV_1_, and FVC effects but did not provide compatible endpoint dispersion data for the older endpoint-value SMD model [11]. Sharma [9] used a three-arm design and reported ANOVA/pairwise comparisons for VO_2_max, PEFR, mMRC, and SF-12; because these outcomes and estimands were not directly compatible with the scale-specific walking test models, they were integrated narratively rather than through ad hoc conversion or control group duplication. Outcome-specific RoB 2 was completed for the principal secondary outcomes, but secondary GRADE certainty remained low to very low because of outcome-specific concerns regarding risk of bias, inconsistency, and imprecision. Several key outcomes were participant-reported in unblinded comparisons, while differences in outcome instruments and estimands further limited comparability. Hospitalizations and respiratory exacerbations could not be graded because comparative results were insufficient. Publication bias and small-study effects cannot be assessed reliably, and safety reporting remains too heterogeneous for a pooled estimate. Source-level trustworthiness concerns were additionally identified for Orooj et al. (2023) [12] and Sharma et al. (2026) [9]. These concerns were not resolved by re-analysis of the published aggregate data and therefore were not corrected or imputed. A structured assessment informed by INSPECT-SR and sensitivity analyses was used to make their influence transparent. Importantly, neither trial contributed to the primary adjusted ISWT meta-analysis, so the primary pooled estimate was unaffected by their exclusion; however, their removal weakens the breadth of supportive narrative and exploratory secondary evidence.

### 4.6. Implications for Future Research

The findings of this review highlight the need for future studies of pulmonary rehabilitation in patients with post-tuberculosis lung disease to be adequately powered, prospectively registered, multicenter randomized controlled trials using standardized diagnostic criteria for PTLD and detailed characterization of the nature and severity of residual pulmonary impairment. Random-sequence generation and allocation concealment should be appropriately implemented and clearly described, and outcome assessment should be blinded where feasible. Trial protocols and statistical analysis plans should be prespecified, with any deviations reported transparently, and primary analyses should follow the intention-to-treat principle. Trial populations should be described in sufficient detail, including baseline characteristics, recruitment periods, study sites, participant flow, and trial registration identifiers. Treatment effects should preferably be reported as baseline-adjusted between-group estimates with 95% confidence intervals, together with sufficient dispersion data to permit inclusion in subsequent quantitative syntheses.

Rehabilitation interventions should be described in sufficient detail to allow replication, including the type and dose of aerobic and resistance exercise, respiratory muscle training where applicable, exercise progression, level of supervision, educational and self-management components, and measures of adherence and intervention fidelity. Core outcomes should include exercise capacity, dyspnea, pulmonary function, health-related quality of life, skeletal muscle function, participation, exacerbations, hospitalizations, and systematically assessed adverse events. Longer-term follow-up is needed to determine the durability of treatment effects. Trials examining home-based rehabilitation and telerehabilitation should also evaluate access, adherence, equity, scalability, and cost-effectiveness, particularly in low- and middle-income countries with a high tuberculosis burden and limited access to pulmonary rehabilitation services. Beyond intervention efficacy, future research should address the integration of PTLD assessment and rehabilitation into routine post-tuberculosis care. Spruijt et al. identified gaps in awareness and clinical follow-up practices concerning post-TB health and well-being, even in a low-tuberculosis-incidence setting [33]. These findings highlight the need for implementation studies evaluating referral pathways, healthcare professional training, access to rehabilitation services, and the feasibility of incorporating functional assessment into routine follow-up. Such research should also examine patient participation, long-term adherence, and the sustainability of rehabilitation programs across different healthcare systems. Further limitations include incomplete original search logs, inability to independently rerun the corrected search formulations, and a targeted rather than comprehensive INSPECT-SR-informed trustworthiness assessment. Unresolved concerns in Orooj et al. (2023) [12] and Sharma et al. (2026) [9] restrict confidence in affected findings.

## 5. Conclusions

Given the very-low-certainty ratings for the principal outcomes, the evidence is very uncertain regarding the effects of structured rehabilitation on exercise capacity and other patient-important outcomes in adults after pulmonary tuberculosis. Peripheral muscle strength was considered an additional exploratory functional outcome rather than a prespecified secondary outcome. The available randomized evidence therefore does not permit a confident estimate of the magnitude of benefit or the optimal program content, dose, duration, device, or delivery model; structured rehabilitation should be interpreted within individualized PTLD care in light of this very low certainty. Two adjusted ISWT trials reported favorable individual effects, and another randomized trial reported a favorable adjusted 6MWD difference. Sharma et al. reported omnibus between-group differences, but important baseline imbalances, unusual control group reporting patterns, and unresolved trustworthiness concerns preclude treating those findings as confirmatory evidence of benefit. Pooled exercise capacity estimates remain very imprecise, and certainty is very low for the principal outcomes. Exploratory estimates involving Orooj et al. (2023) also remain dependent on unresolved source-level concerns. More transparently reported randomized evidence is needed. A two-trial exploratory analysis of inspiratory muscle strength suggested a positive mean change difference, but its wide uncertainty interval and an unresolved discrepancy in the Torres source statistics preclude a firm efficacy conclusion.

## Figures and Tables

**Table 1:** Information sources and search coverage.

Source	Platform	Coverage	Last Search/Status
PubMed/ MEDLINE	NCBI	Database inception onward	13 July 2026 original search; direct update through 28 August 2026
Scopus	Elsevier	Database inception onward	13 July 2026 native search; final targeted update covering Scopus-indexed literature through 5 September 2026
Web of Science Core Collection	Clarivate	Database inception onward	13 July 2026 native search; final targeted update covering Web of Science-indexed literature through 5 September 2026
CENTRAL	Cochrane Library	Database inception onward	13 July 2026 original search; public interface update through 28 August 2026
PEDro	Physiotherapy Evidence Database	Database inception onward	13 July 2026 original search; public interface update checked 28 August 2026
ClinicalTrials.gov	U.S. National Library of Medicine	All years; interventional; all statuses; adult/older adult where applicable	28 August 2026—final registry update completed
WHO ICTRP	World Health Organization	All years and recruitment statuses; source registry records cross-linked	28 August 2026—final cross-registry update completed
Reference lists	Manual screening	Included reports and relevant reviews	Updated through 5 September 2026

Abbreviations: CENTRAL, Cochrane Central Register of Controlled Trials; ICTRP, International Clinical Trials Registry Platform; MEDLINE, Medical Literature Analysis and Retrieval System Online; NCBI, National Center for Biotechnology Information; PEDro, Physiotherapy Evidence Database; WHO, World Health Organization. The original search terminology included “Pulmonary Rehabilitation” labeled as MeSH, although that heading was not valid in 2026. Corrected formulations in Table S2 are post hoc and must not be represented as exact searches originally executed. No complete independent rerun of these corrected strategies was carried out, and their effect on eligible study identification cannot be established. The September Scopus/Web of Science update involved targeted title/DOI verification, not a complete database rerun. Original native export counts and complete search logs were not retained for retrospective verification.

**Table 2:** Key characteristics of the included randomized trial reports.

Report	Setting/Sample	Intervention	Comparator	Primary Exercise Outcome	Interval After Completion of Anti-TB Treatment Before Enrollment
Katagira et al., (2026) [18]	Uganda; 114 randomized; 92 with end-of-intervention ISWT observations	6-week supervised group pulmonary rehabilitation; 12 sessions	Usual care/waiting list rehabilitation	ISWT	Not specified. Participants were required to have completed TB treatment; a history of smear-positive pulmonary TB ≥ 6 months before enrollment was required.
Torres et al., (2025) [17]	Brazil; 30 randomized and analyzed	8-week home-based telerehabilitation; 45 min/day, 5 days/week	Education/minimal guidance without structured rehabilitation	6MWT	Not specified. Participants were enrolled after completing anti-TB treatment.
Orooj et al., (2023) [12]	India; 90 randomized and analyzed	4-week supervised outpatient pulmonary rehabilitation; 16 sessions	Usual activities and medical care	6MWT	>6 months after completion of pharmacological TB treatment.
Orooj et al., (2025) [13]	India; 90 randomized; possible overlap with Orooj (2023) [12]	structured pulmonary rehabilitation; longer follow-up reported	Usual care	6MWT	≥6 months after completion of pharmacological TB treatment.
Akylbekov et al., (2026) [10]	Kyrgyzstan; 114 randomized; 60/53 with discharge outcomes reported	6-week supervised, culturally adapted individualized pulmonary rehabilitation	Usual care	ISWT	Not specified. Completion of TB treatment and confirmation of TB-negative status were required.
Muthukrishnan & Rajadurai, (2026) [11]	India; 120 randomized (60/60); 113 completed 12 weeks	12-week supervised pulmonary rehabilitation + threshold IMT at 30% MIP	Standard pharmacological management + sham IMT	6MWT	Not specified/reported in the accessible publication information.
Sharma et al., (2026) [9]	India; 180 randomized (60/60/60)	1-month device-assisted respiratory rehabilitation: Lung Flute or Acapella twice daily on alternate days; supervised training plus home breathing/thoracic-expansion exercises	Standard post-treatment care; no airway-clearance device	Queens College Step Test-estimated VO_2_max	Not specified. Participants were required to have completed anti-TB treatment and to be clinically stable.

Abbreviations: 6MWT, six-minute walk test; IMT, inspiratory muscle training; ISWT, Incremental Shuttle Walk Test; TB, tuberculosis; VO_2_max, maximal oxygen uptake.

**Table 3:** Selected comparative outcomes reported by the included trials.

Report/Outcome	Comparative Result Reported in the Source
Katagira (2026)—ISWT	Adjusted difference in change: +54.36 m (95% CI 17.22 to 91.51); *p* = 0.004 [18]
Katagira (2026)—MRC dyspnea	Difference in change: −0.28; *p* = 0.030 [18]
Katagira (2026)—CAT	Difference in change: −3.6 (95% CI −6.7 to −0.39); *p* = 0.015 [18]
Katagira (2026)—CCQ total	Difference in change: −0.37 (95% CI −0.68 to −0.06); *p* = 0.004 [18]
Torres (2025)—6MWD	Comparison of change: *p* = 0.133 [17]
Torres (2025)—SGRQ total	Comparison of change: *p* = 0.074 [17]
Torres (2025)—SF-36 physical functioning	Between-group comparison: *p* = 0.002 [17]
Torres (2025)—SF-36 mental health	Between-group comparison: *p* = 0.005 [17]
Orooj (2023)—6MWD	Between-group comparison: *p* = 0.001 [12]
Orooj (2023)—mMRC	Between-group comparison: *p* = 0.005 [12]
Orooj (2025)—6MWD	Post-intervention difference 251.16 m; *p* < 0.001; Sensitivity report because independence from Orooj (2023) is unresolved [13]
Akylbekov (2026)—ISWT	Adjusted difference in change: +123.0 m (95% CI 81.1 to 164.8); *p* < 0.001 [10]
Akylbekov (2026)—MRC dyspnea	Adjusted difference in change: −1.1 (95% CI −1.4 to −0.9); *p* < 0.001 [10]
Akylbekov (2026)—CAT/CCQ	CAT: −8.7 (95% CI −10.5 to −6.9); CCQ: −1.4 (95% CI approximately −1.78 to −1.2); both *p* < 0.001 [10]
Akylbekov (2026)—EQ-5D-5L VAS	Adjusted difference in change: +20.2 (95% CI 15.5 to 24.9); *p* < 0.0001 [10]
Muthukrishnan (2026)—6MWD	Adjusted difference at 12 weeks: +87.4 m (95% CI 74.2 to 100.6); *p* < 0.001 [11]
Muthukrishnan (2026)—MIP	Adjusted difference: +18.6 cmH_2_O (95% CI 15.1 to 22.1); *p* < 0.001 [11]
Muthukrishnan (2026)—SGRQ total	Adjusted difference: −14.8 points (95% CI −17.3 to −12.3); *p* < 0.001 [11]
Muthukrishnan (2026)—spirometry	Adjusted between-group differences: FEV_1_ +0.21 L; FVC +0.29 L [11]
Muthukrishnan (2026)—safety	No serious adverse events reported [11]
Sharma (2026)—VO_2_max	Omnibus three-group ANOVA: F (2,177) = 69.25; *p* < 0.001; η^2^ = 0.44. The source reports Tukey pairwise significance, but baseline VO_2_max differed materially across randomized arms and the post-intervention Lung Flute mean (22.62 mL/kg/min) was below the control mean (32.80 mL/kg/min). Accordingly, this result is not interpreted as uniform device-versus-control benefit [9].
Sharma (2026)—mMRC dyspnea	Three-group ANOVA: F (2,177) = 59.23; *p* < 0.001; η^2^ = 0.40. Lung Flute mMRC decreased from 2.57 to 1.48; Acapella from 2.52 to 2.10 [9].
Sharma (2026)—PEFR	Three-group ANOVA: F (2,177) = 23.24; *p* < 0.001; η^2^ = 0.21. PEFR increased from 248.58 to 281.50 L/min with Lung Flute and from 249.83 to 365.67 L/min with Acapella [9].
Sharma (2026)—SF-12	Acapella SF-12 increased from 34.48 to 44.60 (*p* < 0.001; d = 1.46), whereas Lung Flute decreased from 38.13 to 33.95 (*p* = 0.001; d = 0.57); Trial-level HRQoL findings were therefore mixed across intervention arms [9].

Abbreviations: 6MWD, six-minute walk distance; CAT, COPD Assessment Test; CCQ, Clinical COPD Questionnaire; CI, confidence interval; ISWT, Incremental Shuttle Walk Test; MRC, Medical Research Council; mMRC, modified Medical Research Council; SGRQ, St George’s Respiratory Questionnaire; cmH_2_O, centimeters of water; MIP, maximal inspiratory pressure; PEFR, PEFR, peak expiratory flow rate; SF-12, 12-Item Short Form Health Survey; VO_2_max, maximal oxygen uptake; HRQoL, health-related quality of life.

**Table 4:** RoB 2 assessment for immediate post-intervention exercise capacity.

Report	Overall RoB 2 Judgment for Exercise Capacity	Main Rationale
Katagira et al. (2026) [18]	Some concerns	Missing 6-week ISWT observations in 22/114 randomized participants; otherwise, prespecified primary outcome and blinded assessment were reported.
Torres et al. (2025) [17]	Low	Computer-generated randomization, complete outcome data, and blinded post-intervention assessment were reported.
Orooj et al. (2023) [12]	Some concerns	Limited detail on sequence generation/concealment, assessor blinding not reported, and no accessible prospective statistical analysis plan identified.
Orooj et al. (2025) [13]	High	Concerns regarding randomization reporting, outcome measurement, internal reporting inconsistencies, and selective reporting.
Akylbekov et al. (2026) [10]	Low	Prospective registration/protocol, prespecified ISWT primary endpoint, blinded outcome measurement, and intention-to-treat analysis.
Muthukrishnan & Rajadurai (2026) [11]	Some concerns	Prospective randomized double-blind design with sham IMT and low attrition; allocation concealment details were insufficiently reported in the accessible publication information.
Sharma et al., (2026) [9]	High	Simple lottery allocation without clearly documented concealment; participants and assessors were unblinded; the effort-dependent Queens College Step Test outcome could be influenced by allocation awareness; no prospective protocol/registration was identified.

Abbreviations: ISWT, Incremental Shuttle Walk Test; RoB 2, Cochrane Risk of Bias 2 tool.

**Table 5:** Primary random-effects meta-analysis of adjusted between-group ISWT change.

Report	Outcome	Adjusted Between-Group Effect	95% CI	SE	RoB 2
Katagira et al., (2026) [18]	ISWT change	+54.36 m	17.22 to 91.51	18.95	Some concerns
Akylbekov et al., (2026) [10]	ISWT change	+123.00 m	81.10 to 164.80	21.35	Low
REML random-effects model	ISWT change	+87.98 m	−348.01 to 523.96	—	—

Abbreviations: SE = standard error; CI, confidence interval; ISWT, Incremental Shuttle Walk Test; REML, restricted maximum likelihood; RoB 2, Cochrane Risk of Bias 2 tool.

**Table 6:** Exploratory cross-instrument sensitivity analyses based on end-of-intervention values.

Scenario	Reports Included	Pooled g	95% HK CI	*p*	I^2^
Conservative cross-instrument endpoint SMD	Katagira (2026) [18]; Torres (2025) [17]; Orooj (2023) [12]	1.38	−3.25 to 6.01	0.329	97.4%
Both Orooj reports included	Katagira (2026) [18]; Torres (2025) [17]; Orooj (2023) [12]; Orooj (2025) [13]	1.94	−1.08 to 4.97	0.133	97.4%
Alternative Orooj report retained	Katagira (2026) [18]; Torres (2025) [17]; Orooj (2025) [13]	1.42	−3.40 to 6.24	0.332	97.5%
The first two scenarios include Orooj (2023) [12]; the alternative scenario includes Orooj (2025) [13]. All remain exploratory owing to unresolved source-level concerns.					

Abbreviations: CI, confidence interval; HK, Hartung–Knapp; I^2^, percentage of variability attributable to between-study heterogeneity; SMD, standardized mean difference. Positive Hedges’ g values favor rehabilitation.

**Table 7:** Exploratory meta-analyses of secondary outcomes using independent reports.

Outcome	Independent Reports (k)	Effect Measure	Pooled Estimate	95% Modified HK CI	*p*	I^2^
6MWD	2	MD, m	120.09	−523.84 to 764.01	0.254	81.4%
Dyspnea (MRC/mMRC)	2	Hedges’ g	−0.58	−6.11 to 4.95	0.410	87.6%
SF-36 physical functioning	2	MD, points	32.86	−52.24 to 117.96	0.128	73.4%
SF-36 mental health	2	MD, points	22.63	0.49 to 44.77	0.049	0.0%
All four models include Orooj (2023) [12]. Exclusion leaves only one report per outcome, preventing pooling. MIP source note: Torres change variance reconstructed from within-group 95% Cis [17]; published between-group *p* = 0.266 cannot be reconciled with derived precision. This is an exploratory, non-prespecified analysis.						
MIP (inspiratory muscle strength)	2	MD, cmH_2_O (unadjusted change difference)	+17.75	−10.05 to 45.56	Exploratory	20.6%

Abbreviations: 6MWD, six-minute walk distance; CI, confidence interval; HK, Hartung–Knapp; MD, mean difference; MRC, Medical Research Council; mMRC, modified Medical Research Council; SF-36, 36-Item Short Form Health Survey; SMD, standardized mean difference; I^2^, inconsistency statistic (percentage of variability attributable to between-study heterogeneity), MIP, maximal inspiratory pressure.

**Table 8:** Secondary outcomes not quantitatively pooled.

Outcome	Synthesis	Reason/Principal Result
FEV_1_	Narrative only	Orooj (2023) [12]: no significant independent comparison (*p* = 0.142); Muthukrishnan (2026): adjusted +0.21 L [11].
FVC	Narrative only	Muthukrishnan (2026): adjusted +0.29 L [11]; other reports lacked complete comparable data or contained unusable values.
FEV_1_/FVC	Narrative only	Complete comparable data were unavailable; Orooj (2023) reported FEV_1_/FVC values around/above 100 with unclear scaling or units, and these values were treated as unresolved source-reporting inconsistencies rather than reconstructed [12].
SGRQ/HRQoL	Narrative plus exploratory SF-36 models	Muthukrishnan (2026): SGRQ total −14.8 (95% CI −17.3 to −12.3) [11]; Akylbekov (2026): EQ-5D-5L VAS +20.2 (15.5 to 24.9) [10]. Sharma (2026) reported divergent SF-12 changes across device arms, but interpretation is limited by baseline imbalance and source-reporting concerns [9]; Torres/Orooj SF-36 analyses remain exploratory.
Adverse events	Narrative only	Sparse and non-uniform reporting; no pooled safety estimate. Katagira [18] 12/57 vs. 6/57; Akylbekov [10] 2/61 vs. 1/53; Muthukrishnan [11] reported no serious adverse events.
PEFR	Narrative only	Sharma (2026): significant omnibus three-group difference, F(2,177) = 23.24, *p* < 0.001, η^2^ = 0.21; however, identical control group pre/post values and other source-level reporting concerns limit causal device-versus-control interpretation [9].
Inspiratory muscle strength (MIP)	Narrative only	Muthukrishnan (2026): adjusted between-group MIP +18.6 cmH_2_O (95% CI 15.1 to 22.1) [11]. The Torres public trial record specified MIP/MEP assessment, but the final publication did not provide a numerical MIP result suitable for comparative extraction [17].
Peripheral muscle strength (additional extracted outcome)	Narrative only	Torres (2025): handgrip strength and knee-extension torque favored telerehabilitation; these measures were treated as additional extracted functional outcomes rather than prespecified secondary outcomes [17].

Abbreviations: CAT, COPD Assessment Test; CCQ, Clinical COPD Questionnaire; FEV_1_, forced expiratory volume in one second; FVC, forced vital capacity; MIP, maximal inspiratory pressure; SF-36, 36-Item Short Form Health Survey; SGRQ, St George’s Respiratory Questionnaire; HRQoL, health-related quality of life; CI, confidence interval; EQ-5D-5L, EuroQol 5-Dimension 5-Level questionnaire; VAS, visual analogue scale; SF-12, 12-Item Short Form Health Survey; PEFR, peak expiratory flow rate; MEP, maximal expiratory pressure.

**Table 9:** GRADE Summary of Findings for the primary outcome and principal secondary outcomes.

Outcome	Evidence Base	Comparative Effect/Synthesis	Main GRADE Considerations	Certainty
Exercise capacity (primary)	6 independent RCTs; 648 randomized. Two RCTs (228 randomized) contributed to the adjusted ISWT meta-analysis; four additional RCTs reported 6MWT or Queens College Step Test-estimated VO_2_max outcomes.	Adjusted ISWT MD +87.98 m (95% Hartung–Knapp CI −348.01 to 523.96; I^2^ = 82.7%). Muthukrishnan reported adjusted 6MWD +87.4 m (95% CI 74.2 to 100.6) [11]; Torres had a non-significant between-group 6MWD change comparison [17]; Orooj (2023) [12] reported a favorable between-group difference; Sharma [9] reported a significant three-group VO_2_max difference (F(2,177) = 69.25; *p* < 0.001). Sharma omnibus findings are not confirmatory because of unresolved trustworthiness concerns.	Serious risk of bias (−1), including one high-risk and three some-concerns independent RCTs; very serious inconsistency (−2) because effect magnitudes, instruments, and estimands differed markedly with substantial unexplained heterogeneity; serious imprecision (−1) because the pooled ISWT CI was very wide and crossed the null despite additional favorable trial-level evidence; no serious indirectness; publication bias not assessable.	Very low
Dyspnea	5 RCTs; 618 randomized	Two-report pooled MRC/mMRC Hedges’ g −0.58 (95% modified HK CI −6.11 to 4.95; I^2^ = 87.6%); Akylbekov [10] reported adjusted MRC −1.1 points, Muthukrishnan [11] reported favorable dyspnea outcomes, and Sharma [9] reported a significant three-group mMRC difference (F(2,177) = 59.23; *p* < 0.001). The exploratory pooled result depends on Orooj (2023) [12], while the Sharma [9] dyspnea findings remain difficult to interpret.	Serious risk of bias (−1); serious inconsistency (−1); very serious imprecision (−2); no serious indirectness; publication bias not assessable.	Very low
Health-related quality of life	6 RCTs; 648 randomized	SF-36 physical functioning MD +32.86 (95% modified HK CI −52.24 to 117.96; I^2^ = 73.4%); mental health MD +22.63 (95% modified HK CI 0.49 to 44.77; I^2^ = 0%); newer trials reported EQ-5D-5L VAS +20.2, SGRQ −14.8 points, and mixed SF-12 findings across Sharma [9] intervention arms.	Serious risk of bias (−1); serious inconsistency (−1) across instruments/effects; serious imprecision (−1); no serious indirectness; publication bias not assessable.	Very low
Pulmonary function	4 RCTs; 420 randomized	No pooled estimate. Orooj (2023) [12] reported no significant FEV_1_ between-group difference (*p* = 0.142); Muthukrishnan [11] reported adjusted FEV_1_ +0.21 L and FVC +0.29 L; Sharma [9] reported a significant PEFR difference (F(2,177) = 23.24; *p* < 0.001); measures/estimands were not sufficiently comparable.	Serious risk of bias (−1); serious inconsistency (−1); serious imprecision (−1); no serious indirectness; publication bias not assessable.	Very low
Inspiratory muscle strength (MIP)	2 RCTs; 150 randomized (Torres 30; Muthukrishnan 120)	Muthukrishnan (2026): adjusted between-group MIP +18.6 cmH_2_O (95% CI 15.1 to 22.1) [11]. The Torres trial registry specified MIP/MEP assessment, but the final report did not provide a numerical MIP result suitable for comparative extraction [17]. Exploratory REML/Hartung–Knapp unadjusted MIP change MD +17.75 cmH_2_O (95% CI −10.05 to 45.56; I^2^ approximately 20.6%). Torres +12.1 unadjusted (source *p* = 0.266) [17]; Muthukrishnan +18.6 adjusted (95% CI 15.1 to 22.1) [11].	Inconsistency assessable but highly uncertain (k = 2); very serious imprecision; intervention/estimand differences; outcome-specific RoB 2 to confirm; Torres variance/*p*-value disagreement. Peripheral muscle strength remains a separate additional, non-prespecified outcome. Serious risk of bias (−1) because the contributing RCT raised some concerns; very serious imprecision (−2) because evidence was derived from a single trial; inconsistency could not be assessed; no serious indirectness identified; publication bias not assessable.	Very low (provisional)
Adverse events	4 RCTs; 378 randomized	No pooled estimate. Katagira [18]: 12/57 vs. 6/57 events (two serious, considered unrelated); Torres [17]: no events; Akylbekov [10]: 2 intervention vs. 1 control event; Muthukrishnan: no serious adverse events [11].	Serious risk of bias (−1) because adverse event ascertainment and reporting were not standardized; serious indirectness (−1) because the reported safety outcomes did not represent a uniform adverse event construct across trials, with some studies reporting any adverse events and others reporting only serious or intervention-related events, with incompletely specified definitions and attribution; very serious imprecision (−2) because events were sparse and no reliable comparative safety effect could be estimated; publication bias was not assessable.	Very low
Hospitalizations/respiratory exacerbations	No sufficiently informative comparative result	Not estimable from the eligible randomized reports.	No body of comparative evidence suitable for GRADE.	Not graded
Orooj (2023) [12] affects exploratory outcome estimates; Sharma (2026) [9] omnibus findings cannot establish benefit in view of unresolved baseline and control group reporting concerns.				

Note: GRADE ratings refer to the conservative independent evidence set, excluding Orooj et al. (2025) [13] because participant independence from Orooj et al. (2023) [12] could not be verified. For exercise capacity, the GRADE body includes all six independent RCTs (648 randomized participants), while only the two directly comparable adjusted ISWT trials contributed to the primary scale-specific meta-analysis; Sharma et al. (2026) [9] contributed narratively through Queens College Step Test-estimated VO_2_max. MIP is treated as the prespecified inspiratory muscle strength secondary outcome and is graded separately. Peripheral muscle strength (handgrip and knee-extension torque) is treated as an additional extracted functional outcome and is not presented as prespecified. Unresolved or internally implausible Orooj pulmonary function values were not reconstructed. The exploratory secondary outcome estimates involving Orooj et al. (2023) [12] and narrative Sharma omnibus comparisons should not be treated as confirmatory because of unresolved source-level concerns. For inspiratory muscle strength (MIP), two RCTs (150 participants) are available; the pooled change-score analysis is exploratory and GRADE certainty is provisional pending outcome-specific reassessment. GRADE, Grading of Recommendations Assessment, Development and Evaluation; RCT, randomized controlled trial; ISWT, incremental shuttle walk test; 6MWT, six-minute walk test; 6MWD, six-minute walk distance; MD, mean difference; CI, confidence interval; HK, Hartung–Knapp; I^2^, inconsistency statistic; MRC, Medical Research Council dyspnea scale; mMRC, modified Medical Research Council dyspnea scale; SF-36, 36-Item Short Form Health Survey; EQ-5D-5L, EuroQol 5-Dimension 5-Level questionnaire; VAS, visual analogue scale; SGRQ, St George’s Respiratory Questionnaire; SF-12, 12-Item Short Form Health Survey; FEV_1_, forced expiratory volume in one second; FVC, forced vital capacity; PEFR, peak expiratory flow rate; MIP, maximal inspiratory pressure; MEP, maximal expiratory pressure; REML, restricted maximum likelihood; RoB 2, Risk of Bias 2 tool; VO_2_max, maximal oxygen uptake.

## Data Availability

All data used in this review were derived from published reports and are summarized in the article and Supplementary Materials.

## Supplementary Materials

The following supporting information can be downloaded at: https://www.mdpi.com/article/10.3390/jcm15218397/s1, Table S1. Twelve-month follow-up outcomes reported by Orooj et al. (2025); Table S2. Final database- and registry-specific search strategies; Table S3. Report-level eligibility disposition of the reconciled 76-report screening set; Table S4. Disposition of potentially relevant trial registrations identified or cross-linked during the 28 August 2026 registry update; Table S5. Outcome-specific RoB 2 assessments for principal secondary outcomes included in the Summary of Findings; Table S6. PICOS eligibility verification for Sharma et al. (2026); Table S7. Baseline demographic and clinical characteristics of the two randomized controlled trials included in the primary ISWT meta-analysis; Table S8. Structured source-level trustworthiness assessment of Orooj et al. (2023) and Sharma et al. (2026), informed by INSPECT-SR; Table S9. Deviations from the registered protocol/public record, reasons, and implications for the synthesis (PRISMA 2020 Item 24c); Supplementary Code S1. Python script reproducing the meta-analytic calculations.

## References

[1] World Health Organization (2025). Global Tuberculosis Report 2025.

[2] Migliori G.B., Marx F.M., Ambrosino N., Zampogna E., Schaaf H.S., van der Zalm M.M., Allwood B.W., Byrne A.L., Mortimer K., Wallis R.S. (2021). Clinical standards for the assessment, management and rehabilitation of post-TB lung disease. Int. J. Tuberc. Lung Dis..

[3] Taylor J., Bastos M.L., Lachapelle-Chisholm S., Mayo N.E., Johnston J., Menzies D. (2023). Residual respiratory disability after successful treatment of pulmonary tuberculosis: A systematic review and meta-analysis. eClinicalMedicine.

[4] Gebremedhn A.T., Bobosha K., Adane H.T., Fantaye Y.A., Abebe D.G., Bukate T.A., Mera N.A., Boltena M.T. (2026). Effect of pulmonary rehabilitation on lung function and quality of life in pulmonary tuberculosis survivors with post-tuberculosis lung disease: A systematic review and meta-analysis. BMC Pulm. Med..

[5] Kumar K., Chidambaram A., Venkatesan M., Alahmadi H.I., Alotaibi G.A., Al-Otaibi H.M. (2026). Pulmonary rehabilitation in post-tuberculosis lung disease: A systematic review. Front. Med..

[6] Page M.J., McKenzie J.E., Bossuyt P.M., Boutron I., Hoffmann T.C., Mulrow C.D., Shamseer L., Tetzlaff J.M., Akl E.A., Brennan S.E. (2021). The PRISMA 2020 statement: An updated guideline for reporting systematic reviews. BMJ.

[7] Rethlefsen M.L., Kirtley S., Waffenschmidt S., Ayala A.P., Moher D., Page M.J., Koffel J.B., PRISMA-S Group (2021). PRISMA-S: An extension to the PRISMA Statement for Reporting Literature Searches in Systematic Reviews. Syst. Rev..

[8] Higgins J.P.T., Thomas J., Chandler J., Cumpston M., Li T., Page M.J., Welch V.A. (2024). Cochrane Handbook for Systematic Reviews of Interventions, Version 6.5 (Updated August 2024).

[9] Sharma B., Kumar B., Verma M., Rabra S., Gangania M.K., Goyal H. (2026). Effectiveness of lung flute and acapella devices in pulmonary tuberculosis rehabilitation: A randomized controlled trial. Egypt. J. Bronchol..

[10] Akylbekov A., Orme M.W., Matheson J.A., Richardson M., Taalaibekova A., Mademilov M., Mirzalieva G., Magdieva K., Ozonova A., Erkinbaeva A. (2026). Clinical and cost effectiveness of pulmonary rehabilitation for people with post-tuberculosis lung disease in Kyrgyzstan: A single-blind randomized controlled trial. Ann. Am. Thorac. Soc..

[11] Muthukrishnan P., Rajadurai S. (2026). Pulmonary Rehabilitation and Inspiratory Muscle Training in Post-Tuberculosis Lung Disease: A Randomized Controlled Trial Assessing Functional Capacity, Respiratory Muscle Strength, and Quality of Life. Indian J. Tuberc..

[12] Orooj M., Pant A., Mujaddadi A., Jain M., Ahmed I. (2023). Short-Term Pulmonary Rehabilitation Among Post-Pulmonary Tuberculosis Patients During Coronavirus Disease 2019 Pandemic. Thorac. Res. Pract..

[13] Orooj M., Saraf A., Mujaddadi A., Jain M., Ahmed I., Kuldeep R. (2025). Long-term Effect of Pulmonary Rehabilitation in Pulmonary Tuberculosis Patients. Thorac. Res. Pract..

[14] Wilkinson J., Bero L., Heal C., Flemyng E., Antoniou G.A., Aburrow T., Alfirevic Z., Avenell A., Barbour V., Berghella V. (2026). INSPECT-SR tool for assessing trustworthiness of randomised controlled trials. BMJ.

[15] Sterne J.A.C., Savović J., Page M.J., Elbers R.G., Blencowe N.S., Boutron I., Cates C.J., Cheng H.Y., Corbett M.S., Eldridge S.M. (2019). RoB 2: A Revised Tool for Assessing Risk of Bias in Randomized Trials.

[16] McGuinness L.A., Higgins J.P.T. (2021). Risk-of-bias VISualization (robvis): An R package and Shiny web app for visualizing risk-of-bias assessments. Res. Synth. Methods.

[17] Torres D.F.M., Guimarães F.S., Meireles N.A.S., Cardoso A.P., Migliori G.B., Mello F.C.Q. (2025). Functional capacity and quality of life after telerehabilitation in post-tuberculosis lung disease: A randomized controlled trial. J. Bras. Pneumol..

[18] Katagira W., Orme M.W., Kasiita R., Matheson J.A., Richardson M., Barton A., Nanyonga J., Steiner M.C., Kirenga B., Singh S.J. (2026). Clinical Effectiveness and Cost-Effectiveness of Pulmonary Rehabilitation for People in Uganda with Post-TB Lung Disease: A Randomized Controlled Trial. Chest.

[19] Hartung J., Knapp G. (2001). A refined method for the meta-analysis of controlled clinical trials with binary outcome. Stat. Med..

[20] Röver C., Knapp G., Friede T. (2015). Hartung-Knapp-Sidik-Jonkman approach and its modification for random-effects meta-analysis with few studies. BMC Med. Res. Methodol..

[21] Guyatt G., Oxman A.D., Akl E.A., Kunz R., Vist G., Brozek J., Norris S., Falck-Ytter Y., Glasziou P., DeBeer H. (2011). GRADE guidelines: 1. Introduction—GRADE evidence profiles and summary of findings tables. J. Clin. Epidemiol..

[22] Schünemann H.J., Vist G.E., Higgins J.P.T., Santesso N., Deeks J.J., Glasziou P., Akl E.A., Skoetz N., Guyatt G.H., Higgins J.P.T., Thomas J., Chandler J., Cumpston M., Li T., Page M.J., Welch V.A., Cochrane GRADEing Methods Group (formerly Applicability and Recommendations Methods Group) and the Cochrane Statistical Methods Group (2024). Chapter 14: Completing ‘Summary of findings’ tables and grading the certainty of the evidence. Cochrane Handbook for Systematic Reviews of Interventions, Version 6.5.

[23] Spruit M.A., Singh S.J., Garvey C., ZuWallack R., Nici L., Rochester C., Hill K., Holland A.E., Lareau S.C., Man W.D.-C. (2013). An official American Thoracic Society/European Respiratory Society statement: Key concepts and advances in pulmonary rehabilitation. Am. J. Respir. Crit. Care Med..

[24] Rochester C.L., Alison J.A., Carlin B., Jenkins A.R., Cox N.S., Bauldoff G., Bhatt S.P., Bourbeau J., Burtin C., Camp P.G. (2023). Pulmonary Rehabilitation for Adults with Chronic Respiratory Disease: An Official American Thoracic Society Clinical Practice Guideline. Am. J. Respir. Crit. Care Med..

[25] Singh S.J., Jones P.W., Evans R., Morgan M.D.L. (2008). Minimum clinically important improvement for the incremental shuttle walking test. Thorax.

[26] Silva D.R., Mello F.C.d.Q., Galvão T.S., Dalcolmo M., dos Santos A.P.C., Torres D.d.F.M., Jesus L.A.d.M.C.d., Visca D., Centis R., D’aMbrosio L. (2025). Pulmonary Rehabilitation in Patients with Post-Tuberculosis Lung Disease: A Prospective Multicentre Study. Arch. Bronconeumol..

[27] Rifat M., Silva D.R., Mahmud A.M., Bennoor K.S., D’AMbrosio L., Centis R., Ardesi F., Visca D., Piubello A., Migliori G.B. (2026). Feasibility and efficacy of a population-based pulmonary rehabilitation for post-tuberculosis lung disease in Bangladesh. Int. J. Infect. Dis..

[28] Stenberg B., Drage M., Matewere B., Alvarez-Manon V. (2025). Community-based pulmonary rehabilitation for post-TB lung disease—A programmatic intervention. IJTLD Open.

[29] Kirakosyan O., Verbree H., van der Zalm M.M., Nightingale R., Muñoz-Torrico M., Silva D.R., Singh S.J., Centis R., Duarte R., Akkerman O.W. (2026). Rehabilitation for individuals with post-tuberculosis lung disease. Breathe.

[30] Cox N.S., Dal Corso S., Hansen H., McDonald C.F., Hill C.J., Zanaboni P., Alison J.A., O’Halloran P., Macdonald H., Holland A.E. (2021). Telerehabilitation for chronic respiratory disease. Cochrane Database Syst. Rev..

[31] Pontali E., Silva D.R., Marx F.M., Caminero J.A., Centis R., D’aMbrosio L., Garcia-Garcia J.M., Muhwa J.C., Tiberi S., Migliori G.B. (2022). Breathing Back Better! A State of the Art on the Benefits of Functional Evaluation and Rehabilitation of Post-Tuberculosis and Post-COVID Lungs. Arch. Bronconeumol..

[32] Silva D.R., Pontali E., Kherabi Y., D’aMbrosio L., Centis R., Migliori G.B. (2025). Post-TB Lung Disease: Where Are We to Respond to This Priority?. Arch. Bronconeumol..

[33] Spruijt I., Verhage A., Procee F., Vrubleuskaya N., Mulder C., Akkerman O. (2024). Perceptions and clinical practices associated with post-TB health and well-being in the Netherlands. IJTLD Open.

