# Supplementary material for: Comparative Effectiveness of Rehabilitation Programs for Adults with Post-Tuberculosis Lung Disease: A Systematic Review and Meta-Analysis of Randomized Controlled Trials
Source: J Clin Med. 2026 Oct 28;15(21):8397. doi: 10.3390/jcm15218397 (PMC13764151; doi:10.3390/jcm15218397)
Supplement: Supplementary file 1 [file jcm-15-08397-s001.zip › jcm-4617350-supplementary.pdf]

Journal of
Clinical Medicine

MDPI

Supplementary Table S1 Twelve-month follow-up outcomes reported by Orooj et al., 2025 [13].

Outcome	Pulmonary rehabilitation, n=26	Control, n=34
**6MWD, m**	386.08 ± 64.51	174.72 ± 66.11
**FEV_1, L**	1.45 ± 0.457	1.00 ± 0.377
**FEV_1, % predicted**	24.48 ± 7.12	31.00 ± 6.67
**FVC, L**	1.86 ± 0.50	NR[a]
**FVC, % predicted**	21.32 ± 8.43	28.56 ± 9.0
**FEV_1/FVC**	40.12 ± 14.19	29.60 ± 9.02
**Borg dyspnea**	4.92 ± 0.997	9.52 ± 0.586
**SGRQ impact**	41.92 ± 16.91	95.80 ± 2.29
**SGRQ activity**	55.37 ± 14.02	78.00 ± 6.87
**SGRQ symptoms**	51.64 ± 6.95	84.92 ± 9.09
**SGRQ total**	32.40 ± 12.69	86.88 ± 8.33

Data are presented as mean ± standard deviation. 6MWD, six-minute walk distance; FEV_1, forced expiratory volume in one second; FVC, forced vital capacity; SGRQ, St George's Respiratory Questionnaire; NR, not reliably extractable. [a] The control-group FVC value at 12 months was reported as 1.48 ± 34 in the original publication. Because the reported dispersion value could not be reliably interpreted, this result was treated as unusable for quantitative extraction (NR). No correction or imputation was applied. Clarification was requested from the corresponding author, but no response had been received by the time of manuscript revision. These data are presented descriptively and were not included in the primary independent quantitative synthesis because participant independence from Orooj et al., 2023 [12] could not be verified.

Supplementary Table S2. Documented search formulations with post hoc corrections identified separately. Historical exact executed strings should only be labelled as such where supported by the original search log; the corrected strings were not independently rerun. A complete source-by-source rerun was not performed, and unavailable database-specific retrieval counts were not retrospectively reconstructed.

Source	Exact search strategy / search approach
PubMed/MEDLINE	(("Tuberculosis, Pulmonary"[Mesh] OR "pulmonary tuberculosis"[tiab] OR "post-tuberculosis lung disease"[tiab] OR "post tuberculosis lung disease"[tiab] OR PTLD[tiab] OR "post-TB"[tiab] OR "tuberculosis sequela*"[tiab] OR "respiratory sequela*"[tiab]) AND ("Respiratory Therapy"[Mesh] OR "Breathing Exercises"[Mesh] OR "Exercise Therapy"[Mesh] OR "pulmonary rehabilitation"[tiab] OR "respiratory rehabilitation"[tiab] OR physiotherap*[tiab] OR "physical therap*"[tiab] OR "exercise training"[tiab] OR "aerobic exercise"[tiab] OR "resistance training"[tiab] OR "respiratory muscle training"[tiab] OR "breathing exercise*"[tiab] OR telerehabilitation[tiab]) AND (randomized controlled trial[pt] OR controlled clinical trial[pt] OR random*[tiab] OR trial[tiab]))
Scopus	TITLE-ABS-KEY(("pulmonary tuberculosis" OR "post-tuberculosis lung disease" OR "post tuberculosis lung disease" OR PTLD OR "post-TB" OR "tuberculosis sequela*" OR "respiratory sequela*") AND ("pulmonary rehabilitation" OR "respiratory rehabilitation" OR physiotherap* OR "physical therap*" OR "exercise training" OR "aerobic exercise" OR "resistance training" OR "respiratory muscle training" OR "breathing exercise*" OR telerehabilitation) AND (random* OR trial OR "controlled trial"))<br>Final targeted update through 5 September 2026 using the same TITLE-ABS-KEY concepts; recent candidate records were verified by title/DOI. Sharma et al. 2026 [9] was identified as eligible. Exact authenticated-interface export yield was not retained in the working log.
Web of Science Core Collection	TS=(("pulmonary tuberculosis" OR "post-tuberculosis lung disease" OR "post tuberculosis lung disease" OR PTLD OR "post-TB" OR "tuberculosis sequela*" OR "respiratory sequela*") AND ("pulmonary rehabilitation" OR "respiratory rehabilitation" OR physiotherap* OR "physical therap*" OR "exercise training" OR "aerobic exercise" OR "resistance training" OR "respiratory muscle training" OR "breathing exercise*" OR telerehabilitation) AND (random* OR trial OR

Source	Exact search strategy / search approach
	"controlled trial")) Final targeted update through 5 September 2026 using the same TS concepts; recent candidate records were verified by title/DOI. Sharma et al. 2026 [9] was identified as eligible. Exact authenticated-interface export yield was not retained in the working log.
CENTRAL	([mh "Tuberculosis, Pulmonary"] OR "pulmonary tuberculosis":ti,ab,kw OR "post-tuberculosis lung disease":ti,ab,kw OR "post tuberculosis lung disease":ti,ab,kw OR PTLD:ti,ab,kw OR "post-TB":ti,ab,kw OR "tuberculosis sequela*":ti,ab,kw) AND ([mh "Respiratory Therapy"] OR [mh "Breathing Exercises"] OR [mh "Exercise Therapy"] OR "pulmonary rehabilitation":ti,ab,kw OR "respiratory rehabilitation":ti,ab,kw OR physiotherap*:ti,ab,kw OR "physical therap*":ti,ab,kw OR "exercise training":ti,ab,kw OR "aerobic exercise":ti,ab,kw OR "resistance training":ti,ab,kw OR "respiratory muscle training":ti,ab,kw OR "breathing exercise*":ti,ab,kw OR telerehabilitation:ti,ab,kw) AND (random*:ti,ab,kw OR trial:ti,ab,kw)
PEDro	Advanced-search free-text queries adapted to PEDro: (1) tuberculosis rehabilitation; (2) tuberculosis exercise; (3) post-tuberculosis; with Method = clinical trial where the interface permits.
ClinicalTrials.gov	Final update 28 August 2026. Query concepts: (post-tuberculosis OR "post tuberculosis" OR post-TB OR PTLD OR "tuberculosis sequelae" OR "post-tuberculosis bronchiectasis") AND ("pulmonary rehabilitation" OR rehabilitation OR exercise OR physiotherapy OR telerehabilitation OR "respiratory muscle training" OR "breathing exercises" OR "respiratory muscle stretching"). Study type: Interventional; all recruitment statuses; all years; Adult/Older Adult where applicable. Records were cross-linked to publications using NCT/secondary IDs, titles, investigators, and study characteristics.
WHO ICTRP	Final update 28 August 2026. Standard/advanced search using the same population and intervention concepts: (post-tuberculosis OR "post tuberculosis" OR post-TB OR PTLD OR "tuberculosis sequelae") AND ("pulmonary rehabilitation" OR rehabilitation OR exercise OR physiotherapy OR telerehabilitation OR "respiratory muscle training" OR "breathing exercises" OR "respiratory muscle stretching"). All recruitment statuses and all years. Bridged records from primary registries were cross-linked and de-duplicated. At the search date, the portal reported source imports through 10 August 2026 for ClinicalTrials.gov/ChiCTR and 3 August 2026 for CTRI.

Note: These revised controlled-vocabulary formulations were prepared post hoc; they are not verified verbatim reproductions of the originally executed searches. No new yields or screening results are claimed. Counts reported by the reviewer must not be attributed to searches performed by the authors.

Supplementary Table S3. Report-level disposition of the 76 candidate reports assessed for eligibility.

No.	Database	Report title	Decision	Primary reason
40	Scopus	A pre–post intervention study of pulmonary rehabilitation for adults with post-tuberculosis lung disease in Uganda	Excluded	Non-randomized, observational, qualitative, or otherwise ineligible primary study
45	Scopus	A rare case report on post tuberculosis sequelae with right ventricular failure in young adult and the positive impact of cardio-pulmonary rehabilitation on functional independence	Excluded	Case report or case study
52	Cochrane	A study on the effect of home rehabilitation based on consolidation treatment period on pulmonary function and inflammatory factors in patients with pulmonary tuberculosis	Excluded	Wrong population or timing (active TB, COPD, pediatric population, or rehabilitation before treatment completion)

No.	Database	Report title	Decision	Primary reason
193	Web ofScience	Clinical and cost-effectiveness of pulmonary rehabilitation for people with post- tuberculosis lung disease in Uganda: a randomized controlled trial	Included	Eligible randomized controlled trial report
271	Web ofScience	Culturally adapted pulmonary rehabilitation for adults living with post-tuberculosis lung disease in Kyrgyzstan: protocol for a randomized controlled trial with blinded outcome measures	Excluded as protocol / linked to update publication	Protocol in original 76-report set; final RCT results publication (Akylbekov et al. 2026) identified in the 28 August 2026 update and integrated separately [10].
290	Web ofScience	Development and evaluation of an early rehabilitation nursing program for patients with pulmonary tuberculosis	Excluded	Wrong population or timing (active TB, COPD, pediatric population, or rehabilitation before treatment completion)
357	Web ofScience	Effect of home-based pulmonary rehabilitation on ventilation dynamics and small airway dysfunction in people with post-tuberculosis lung disease	Excluded	Non-randomized, observational, qualitative, or otherwise ineligible primary study
362	PubMed	Effect of pulmonary rehabilitation in patients with post-tuberculosis sequelae with functional limitation	Excluded	Non-randomized, observational, qualitative, or otherwise ineligible primary study
363	Web ofScience	Effect of pulmonary rehabilitation on functional capacity in individuals treated for pulmonary tuberculosis: a systematic review protocol	Excluded	Review, guideline/statement, commentary/letter, or correction
364	Scopus	Effect of pulmonary rehabilitation on lung function and quality of life in pulmonary tuberculosis survivors with post-tuberculosis lung disease: a systematic review and meta-analysis	Excluded	Review, guideline/statement, commentary/letter, or correction
379	WebOfScience	Effectiveness of pulmonary rehabilitation on functional exercise capacity & health-related quality of life (HRQOL) among individuals with post tuberculosis lung disease: A multicentric pre & post-interventional study	Excluded	Non-randomized, observational, qualitative, or otherwise ineligible primary study
406	Scopus	Efficacy of early structured pulmonary rehabilitation program in pulmonary function, exercise capacity, and health-related quality of life for patients with post-tubercular sequelae: A pilot study	Excluded	Wrong timing: pulmonary rehabilitation was delivered while anti-tuberculosis treatment was still ongoing, whereas this review required completion of anti-tuberculosis treatment.
424	Cochrane	Endurance Training in Patients With Post-TB Lung Disease	Excluded	Protocol or trial-registry record without an eligible published results report
450	Scopus	Exercise training for the improvement of exercise performance of patients with pulmonary tuberculosis sequelae	Excluded	Non-randomized, observational, qualitative, or otherwise ineligible primary study
477	WebOfScience	From Health-Related Quality of Life to Functional Aerobic Capacity: The Benefits of Pulmonary Rehabilitation in Colombian Patients with Sequelae of Pulmonary Tuberculosis	Excluded	Non-randomized, observational, qualitative, or otherwise ineligible primary study

No.	Database	Report title	Decision	Primary reason
478	WebOfScience	Functional capacity and quality of life after telerehabilitation in post-tuberculosis lung disease: a randomized controlled trial	Included	Eligible randomized controlled trial report
536	Cochrane	Home-Based Lung Rehabilitation for Post Tuberculosis Patients	Excluded	Protocol or trial-registry record without an eligible published results report
570	WebOf-Science	Impact of six week hospital based Pulmonary Rehabilitation programme in patients with post Pulmonary Tuberculosis sequelae: Initial results from a hospital based longitudinal study	Excluded	Non-randomized, observational, qualitative, or otherwise ineligible primary study
596	WebOf-Science	Inpatient pulmonary rehabilitation followed by Tele-Rehabilitation in a patient with Post Tubercular sequelae: A case study	Excluded	Case report or case study
599	Scopus	Integrating pulmonary rehabilitation into post-tuberculosis care: Evidence-based rationale for policy change in high tuberculosis burden countries	Excluded	Review, guideline/statement, commentary/letter, or correction
615	Scopus	Is there a rationale for pulmonary rehabilitation following successful chemotherapy for tuberculosis?	Excluded	Review, guideline/statement, commentary/letter, or correction
633	WebOf-Science	Long-term Effect of Pulmonary Rehabilitation in Pulmonary Tuberculosis Patients	Included	Eligible randomized controlled trial report
692	Scopus	National Research Institute of Tuberculosis and Lung Disease (NRITLD) Implementation Protocol of an Exercise Training Program in Pulmonary Rehabilitation of Patients with COPD	Excluded	Wrong population or timing (active TB, COPD, pediatric population, or rehabilitation before treatment completion)
875	WebOf-Science	Pulmonary rehabilitation for post-TB lung disease led by TB survivors	Excluded	Non-randomized, observational, qualitative, or otherwise ineligible primary study
876	WebOf-Science	Pulmonary Rehabilitation for Post-Tuberculosis Lung Disease	Excluded	Review, guideline/statement, commentary/letter, or correction
884	WebOf-Science	Pulmonary Rehabilitation in Patients with Chronic Lung Impairment from Pulmonary Tuberculosis	Excluded	Non-randomized, observational, qualitative, or otherwise ineligible primary study
885	WebOf-Science	Pulmonary Rehabilitation in Patients With Post-Tuberculosis Lung Disease: A Prospective Multicentre Study	Excluded	Non-randomized, observational, qualitative, or otherwise ineligible primary study
886	WebOf-Science	Pulmonary Rehabilitation in Post Tuberculosis Patients: A Longitudinal Cohort Study	Excluded	Non-randomized, observational, qualitative, or otherwise ineligible primary study
887	WebOf-Science	Pulmonary rehabilitation in severe post-tuberculosis sequelae - case presentation	Excluded	Case report or case study
889	WebOf-Science	Pulmonary rehabilitation is effective in patients with tuberculosis pulmonary sequelae	Excluded	Non-randomized, observational, qualitative, or otherwise ineligible primary study
908	Scopus	Quality of life and influencing factors in patients with post-tuberculosis lung disease after rifampicin-resistant tuberculosis; [利福平耐药结核后肺疾病患者生存质量及其影响因素分析]	Excluded	No eligible structured rehabilitation intervention
923	Scopus	Rehabilitation for individuals with post-tuberculosis lung disease	Excluded	Review, guideline/statement, commentary/letter, or correction
924	Cochrane	Rehabilitation of People With Post-tuberculosis Lung Disease	Excluded	Protocol or trial-registry record without an eligible published

No.	Database	Report title	Decision	Primary reason
				results report
925	WebOf-Science	Rehabilitation programs in decrease chest expansion and low cardiorespiratory endurance in secondary spontaneous pneumothorax due to history of pulmonary tuberculosis with hospital readmission	Excluded	Non-randomized, observational, qualitative, or otherwise ineligible primary study
991	WebOf-Science	Short-Term Pulmonary Rehabilitation Among Post-Pulmonary Tuberculosis Patients During Coronavirus Disease 2019 Pandemic	Included	Eligible randomized controlled trial report
992	PEDro	Short-term pulmonary rehabilitation among post-pulmonary tuberculosis patients during coronavirus disease 2019 pandemic [with consumer summary]	Excluded	Duplicate/companion database record of an included report
1018	WebOf-Science	Study protocol for a randomised controlled trial assessing the impact of pulmonary rehabilitation on maximal exercise capacity for adults living with post-TB lung disease: Global RECHARGE Uganda	Excluded	Protocol or trial-registry record without an eligible published results report
1122	WebOf-Science	The role of pulmonary rehabilitation in patients with tuberculosis sequelae	Excluded	Review, guideline/statement, commentary/letter, or correction
1132	WebOf-Science	To Determine the Effectiveness of Pulmonary Rehabilitation on Functional Exercise Capacity and Health-related Quality of Life (HRQOL) in Patients With Post Tuberculosis Lung Disease: Pre-and Post-Interventional Cohort Study	Excluded	Non-randomized, observational, qualitative, or otherwise ineligible primary study
373	WebOf-Science	Effectiveness of a home-based pulmonary rehabilitation programme in pulmonary function and health-related quality of life for patients with pulmonary tuberculosis: a pilot study	Excluded	Non-randomized, observational, qualitative, or otherwise ineligible primary study
407	WebOf-Science	Efficacy of mindful meditation in adjunct to a pulmonary rehabilitation program in improving functional capacity and quality of life in post-treated pulmonary tuberculosis patients: A randomized controlled trial (Study Protocol)	Excluded	Protocol or trial-registry record without an eligible published results report
906	WebOf-Science	Qualitative evaluation of culturally adapted pulmonary rehabilitation (PR) for people with post-tuberculosis lung disease (pTBLD) in Kyrgyz Republic	Excluded	Non-randomized, observational, qualitative, or otherwise ineligible primary study
1211	WebOf-Science	What should Pulmonary Rehabilitation (PR) look like for people with post-tuberculosis lung disease (PTBLD) in Kyrgyzstan?	Excluded	Non-randomized, observational, qualitative, or otherwise ineligible primary study
1212	WebOf-Science	What should pulmonary rehabilitation look like for people living with post-tuberculosis lung disease in the Bishkek and Chui region of the Kyrgyz Republic? A qualitative exploration	Excluded	Non-randomized, observational, qualitative, or otherwise ineligible primary study
41	Scopus	A prospective study on quality of life in patients with pulmonary tuberculosis at a tertiary care hospital in Kashmir, Northern India	Excluded	Non-randomized, observational, qualitative, or otherwise ineligible primary study
43	WebOf-Science	A qualitative study on the development of pulmonary rehabilitation for patients with chronic lung disease in Kampala, Uganda	Excluded	Non-randomized, observational, qualitative, or otherwise ineligible primary study
97	Scopus	An official American thoracic society/European respiratory society statement: Key concepts and advances in pulmonary rehabilitation	Excluded	Review, guideline/statement, commentary/letter, or correction

https://doi.org/10.3390/jcm15218397

No.	Database	Report title	Decision	Primary reason
111	Scopus	Applicability of the chronic obstructive pulmonary disease assessment test as a measure of health status in patients with sequelae of pulmonary tuberculosis	Excluded	Non-randomized, observational, qualitative, or otherwise ineligible primary study
117	WebOf-Science	Assessing Quality of Life in Patients with New and Recurrent Pulmonary Tuberculosis: A Cross-Sectional Study from Garut, Indonesia	Excluded	Non-randomized, observational, qualitative, or otherwise ineligible primary study
151	WebOf-Science	Breathing Back Better! A State of the Art on the Benefits of Functional Evaluation and Rehabilitation of Post-Tuberculosis and Post-COVID Lungs	Excluded	Review, guideline/statement, commentary/letter, or correction
165	WebOf-Science	Cavitary lung lesions and quality of life after TB	Excluded	Non-randomized, observational, qualitative, or otherwise ineligible primary study
174	Scopus	Characteristics of rehabilitation programs for chronic respiratory diseases in Asia: A scoping review	Excluded	Review, guideline/statement, commentary/letter, or correction
183	WebOf-Science	Chronic lung disease in adult recurrent tuberculosis survivors in Zimbabwe: a cohort study	Excluded	Non-randomized, observational, qualitative, or otherwise ineligible primary study
198	Scopus	Clinical characteristics of patients with post-tuberculosis bronchiectasis: Findings from the KMBARC registry	Excluded	Non-randomized, observational, qualitative, or otherwise ineligible primary study
206	WebOf-Science	Clinical Profile and Quality of Life of Patients With Post-pulmonary Tuberculosis Sequelae Presenting to a Tertiary Care Hospital	Excluded	Non-randomized, observational, qualitative, or otherwise ineligible primary study
244	WebOf-Science	Complex respiratory and motor rehabilitation program in a patient with post-pulmonary tuberculosis conditions and multiple sequelae after SARS-CoV-2 infection: case report	Excluded	Case report or case study
410	WebOf-Science	Efficacy of Pulmonary Rehabilitation in Pulmonary Tuberculosis Sequelae with Cystic Bronchiectasis and Pulmonary Hypertension	Excluded	Case report or case study
564	Scopus	Impact of post-pulmonary tuberculosis sequelae and its management in a tertiary care facility in South India	Excluded	Non-randomized, observational, qualitative, or otherwise ineligible primary study
684	WebOf-Science	Mycobacterium tuberculosis and Pulmonary Rehabilitation: From Novel Pharmacotherapeutic Approaches to Management of Post-Tuberculosis Sequelae	Excluded	Review, guideline/statement, commentary/letter, or correction
764	WebOf-Science	Peripheral Muscle Strength and Pulmonary Function Negatively Impact Functional Capacity in Patients With Post-Tuberculosis Lung Disease: A Cross-Sectional Study	Excluded	Non-randomized, observational, qualitative, or otherwise ineligible primary study
938	Cochrane	Respiratory Muscle Stretching for Improving Chest Expansion and Dyspnea in Post-Tuberculosis Bronchiectasis	Excluded	Protocol or trial-registry record without an eligible published results report
1015	WebOf-Science	Structured Pulmonary Rehabilitation in a Patient with Post Tuberculosis Bronchiectasis and Endobronchial Carcinoid	Excluded	Case report or case study
744	Cochrane	Paediatric Post-TB Pulmonary Rehab Study	Excluded	Wrong population or timing (active TB, COPD, pediatric population, or rehabilitation before treatment completion)

No.	Database	Report title	Decision	Primary reason
792	WebOf-Science	Post-Tuberculosis (TB) Treatment: The Role of Surgery and Rehabilitation	Excluded	Review, guideline/statement, commentary/letter, or correction
653	WebOf-Science	Managing severe tuberculosis and its sequelae: from intensive care to surgery and rehabilitation	Excluded	Review, guideline/statement, commentary/letter, or correction
1120	WebOf-Science	The role of neutrophil response in lung damage and post-tuberculosis lung disease: a translational narrative review	Excluded	Review, guideline/statement, commentary/letter, or correction
366	WebOf-Science	Effect of Telerehabilitation in a Patient with Pulmonary Tuberculosis: A Case Study	Excluded	Case report or case study
881	Scopus	Pulmonary rehabilitation in multi-drug resistant tuberculosis (TB MDR): A case report; [Rehabilitación pulmonar en tuberculosis multirresistente (TB-MDR): Informe de un caso]	Excluded	Case report or case study
30	WebOf-Science	A development study of pulmonary rehabilitation for patients with chronic lung disease in Uganda	Excluded	Non-randomized, observational, qualitative, or otherwise ineligible primary study
258	Scopus	Correlations between exercise oxygen consumption, lung function, image findings, and quality of life in adults with post-tuberculosis lung disease	Excluded	Non-randomized, observational, qualitative, or otherwise ineligible primary study
786	Scopus	Post-pulmonary tuberculosis lung function: a systematic review and meta-analysis	Excluded	Review, guideline/statement, commentary/letter, or correction
787	WebOf-Science	Post-pulmonary tuberculosis lung function: a systematic review and meta-analysis (vol 13, pg e1020, 2025)	Excluded	Review, guideline/statement, commentary/letter, or correction
606	WebOf-Science	Interventions to prevent post-tuberculosis sequelae: a systematic review and meta-analysis	Excluded	Review, guideline/statement, commentary/letter, or correction
1191	WebOf-Science	Unveiling Silent Consequences: Impact of Pulmonary Tuberculosis on Lung Health and Functional Wellbeing after Treatment	Excluded	Non-randomized, observational, qualitative, or otherwise ineligible primary study
1035	WebOf-Science	Synergistic effects of physiotherapy and diaphragmatic stretching in post-tuberculosis pleural effusion management: A case report	Excluded	Case report or case study
1064	Cochrane	The Benefits of Diaphragmatic Stretching for Lung Health After TB	Excluded	Registry record subsequently linked to an August 2026 randomized publication; excluded because the intervention was a same-day single-session diaphragmatic stretching technique rather than a structured repeated rehabilitation program.

Note: The 76-report set corresponds to the reconciled full-text/eligibility screening log (44 records from Sheet1 and 32 from Sheet2). No report remained classified as not retrieved after reconciliation. The duplicate PEDro/consumer-summary record of Orooj et al. 2023 [12] was excluded as a duplicate/companion database record; the published trial report was retained.

Supplementary Table S4. Disposition of potentially relevant trial registrations identified or cross-linked during the final ClinicalTrials.gov/WHO ICTRP update (28 August 2026).

Trial ID	Trial / short title	Status at update	Linkage / decision
ISRCTN18256843	Global RECHARGE Uganda	Completed	Linked to Katagira et al. 2026 [18]; already included.

Trial ID	Trial / short title	Status at update	Linkage / decision
NCT04844502	PTLD telerehabilitation trial	Completed	Linked to Torres et al. 2025/2026 [17]; already included.
CTRI/2022/03/041223	Short-term pulmonary rehabilitation during COVID-19	Completed/ published	Linked to Orooj et al. 2023 [12]; already included.
CTRI/2022/08/045006	Long-term outpatient pulmonary rehabilitation	Published	Linked to Orooj et al. 2025 [13]; sensitivity report because population independence remains unresolved.
CTRI/2023/08/056742	PR + inspiratory muscle training in PTLD	Published 1 Aug 2026	New eligible RCT report identified: Muthukrishnan and Rajadurai 2026; integrated into qualitative/adjusted outcome synthesis. Not merged into endpoint-value SMD because compatible group dispersion data were unavailable [11].
NCT06127641	Rehabilitation of People With Post-tuberculosis Lung Disease	Ongoing/recruiting	Potentially eligible RCT design; no eligible randomized results report identified at update.
NCT06047795	Endurance Training in Patients With Post-TB Lung Disease	Completed	No eligible published results report identified at update.
NCT06597409	Respiratory Muscle Stretching in Post-TB Bronchiectasis	Completed	No eligible published results report identified at update.
NCT06722547	Respiratory Muscle Stretching + standard PR in post-TB bronchiectasis	Completed	No eligible published results report identified at update.
NCT07097961	Paediatric Post-TB Pulmonary Rehab Study	Registered	Excluded: pediatric population (6-17 years); single-arm feasibility design.
NCT07602673	Pulmonary Rehabilitation in African Countries (EQUI-RESP-AFRICA)	Ongoing	Mixed chronic respiratory disease population including post-TB; no PTLD-specific eligible results report identified.
NCT07731009	AFTER-TB: mechanisms, biomarkers and pulmonary rehabilitation	Not yet recruiting / ongoing	No results report; retain as ongoing potentially relevant study.
CTRI/2022/03/040830	Home-Based Lung Rehabilitation for Post Tuberculosis Patients	Completed	Registry-only; no eligible published results report identified at update.
CTRI/2025/03/083611	Mindful meditation adjunct to pulmonary rehabilitation	Protocol / ongoing	Protocol only; no results report identified.
CTRI/2025/07/090423	Diaphragmatic stretching in post-pulmonary TB sequelae	Published 20 Aug 2026	Randomized publication identified but excluded: same-day single-session stretching did not meet the prespecified structured rehabilitation definition.
ChiCTR2200058213	Home rehabilitation during TB consolidation treatment	Completed/ registered	Excluded: rehabilitation occurred during consolidation/ongoing anti-TB treatment, before treatment completion.
ISRCTN11122503	Culturally adapted pulmonary rehabilitation for adults with PTLD in Kyrgyzstan	Completed / final RCT published 2026	New eligible RCT report identified: Akylbekov et al. 2026; final results identified in the update and integrated into the primary adjusted ISWT synthesis [10].

Registry/update note: WHO ICTRP aggregates records from multiple primary registries. After deduplication and publication linkage, 17 unique potentially relevant registrations are listed above; these do not represent the raw unfiltered registry-search yield. Two eligible published RCT reports

were identified through the 28 August 2026 bibliographic/registry update and linked to registrations: Akylbekov et al. 2026 (ISRCTN11122503) [10] and Muthukrishnan and Rajadurai 2026 (CTRI/2023/08/056742) [11]. Sharma et al. 2026 [9] was identified through the final bibliographic update to 5 September 2026; no trial-registration identifier was reported in the publication.
Supplementary

Supplementary Table S5. Outcome-specific RoB 2 judgments for principal secondary outcomes in the conservative independent evidence set.

Outcome	Report	D1	D2	D3	D4	D5	Overall
Dyspnea	Katagira 2026 [18]	Low	Low	Some concerns	Some concerns	Low	Some concerns
Dyspnea	Orooj 2023 [12]	Some concerns	Low	Low	Some concerns	Some concerns	Some concerns
Dyspnea	Akylbekov 2026 [10]	Low	Low	Low	Some concerns	Low	Some concerns
Dyspnea	Muthukrishnan 2026 [11]	Some concerns	Low	Low	Some concerns	Low	Some concerns
Dyspnea	Sharma 2026 [9]	Some concerns	Low	Low	High	Some concerns	High
Health-related quality of life	Katagira 2026 [18]	Low	Low	Some concerns	Some concerns	Low	Some concerns
Health-related quality of life	Torres 2025 [17]	Low	Low	Low	Some concerns	Low	Some concerns
Health-related quality of life	Orooj 2023 [12]	Some concerns	Low	Low	Some concerns	Some concerns	Some concerns
Health-related quality of life	Akylbekov 2026 [10]	Low	Low	Low	Some concerns	Low	Some concerns
Health-related quality of life	Muthukrishnan 2026 [11]	Some concerns	Low	Low	Some concerns	Low	Some concerns
Health-related quality of life	Sharma 2026 [9]	Some concerns	Low	Low	High	Some concerns	High
Pulmonary function	Torres 2025 [17]	Low	Low	Low	Low	Low	Low
Pulmonary function	Orooj 2023 [12]	Some concerns	Low	Low	Some concerns	Some concerns	Some concerns
Pulmonary function	Muthukrishnan 2026 [11]	Some concerns	Low	Low	Low	Low	Some concerns
Pulmonary function (PEFR)	Sharma 2026 [9]	Some concerns	Low	Low	Low	Some concerns	Some concerns
Peripheral muscle strength	Torres 2025 [17]	Low	Low	Low	Low	Low	Low
Adverse events	Katagira 2026 [18]	Low	Low	Low	Some concerns	Low	Some concerns
Adverse events	Torres 2025 [17]	Low	Low	Low	Some concerns	Low	Some concerns

https://doi.org/10.3390/jcm15218397

Outcome	Report	D1	D2	D3	D4	D5	Overall
Adverse events	Akylbekov 2026 [10]	Low	Low	Low	Some concerns	Low	Some concerns
Adverse events	Muthukrishnan 2026 [11]	Some concerns	Low	Low	Some concerns	Low	Some concerns

Note: Judgment rationale: D1 reflects trial-specific randomization and allocation-concealment information. D2 was low where no important deviations affecting the effect of assignment were identified. D3 was rated some concerns for Katagira participant-reported outcomes because 22/114 participants lacked six-week observations; safety counts were reported for both randomized groups. D4 was rated some concerns for participant-reported dyspnea and quality-of-life outcomes when participants knew whether they received rehabilitation. Sharma et al. 2026 [9] was rated high for dyspnea and HRQoL measurement because neither participants nor assessors were blinded and both outcomes were susceptible to allocation awareness; its PEFR result raised some concerns overall because randomization concealment and prospective reporting information were limited. D5 was low when prospective registration/protocol information supported the selected result; Orooj 2023 retained some concerns because a sufficiently detailed prospective analysis plan was unavailable, and Sharma retained some concerns because no prospective protocol or registration was identified. Orooj 2025 was excluded because participant independence from Orooj 2023 could not be established. No adverse-event result was reported by Orooj 2023, and Sharma did not report a distinct safety outcome. D1: randomization process; D2: deviations from intended interventions; D3: missing outcome data; D4: measurement of the outcome; D5: selection of the reported result.

Supplementary Table S6. PICOS eligibility verification for Sharma et al. 2026 [9]. The INSPECT-SR-informed screening presented for Orooj 2023 and Sharma 2026 was targeted and not systematically performed for every trial. Absence of a targeted assessment cannot establish the trustworthiness of the remaining reports.

PICOS element	Prespecified review criterion	Sharma et al. 2026 evidence	Judgment
**Population**	Adults aged ≥18 years with completed anti-tuberculosis treatment and persistent respiratory symptoms and/or residual structural, physiological, or functional impairment attributable to previous pulmonary tuberculosis; active pulmonary tuberculosis excluded; no minimum post-treatment interval required.	AFB-negative adults aged 18–70 years who had completed anti-TB treatment and were clinically stable. Baseline mMRC dyspnea remained substantial in all three groups (control 2.95 ± 0.85; Lung Flute 2.57 ± 1.14; Acapella 2.52 ± 1.16), documenting ongoing post-treatment respiratory symptoms. AFB-positive disease and major chronic respiratory comorbidities were excluded.	**Eligible**
**Intervention**	Structured rehabilitation containing a planned exercise or respiratory-training component; supervised, partially supervised, home-based, or telerehabilitation formats were eligible.	Lung Flute or Acapella was prescribed twice daily on alternate days for one month. Participants received supervised device training, directly monitored sessions, adherence follow-up, and supplementary home breathing and thoracic-expansion exercises, constituting a structured and reproducible respiratory-rehabilitation intervention.	**Eligible**
**Comparator**	Usual care, no structured rehabilitation, waiting-list control, or minimal education without a structured exercise-based rehabilitation program.	The control group received standard post-treatment clinical care without an airway-clearance device, including routine physician follow-up, nutritional counselling, routine breathing exercises, and clinically indicated symptomatic medication. No structured, progressive, or reproducible rehabilitation protocol comparable to the device arms was described for the control group.	**Eligible as usual care**

PICOS element	Prespecified review criterion	Sharma et al. 2026 evidence	Judgment
**Outcomes**	Exercise capacity immediately after the intervention was the primary outcome; pulmonary function, dyspnea, health-related quality of life, muscle strength, adverse events, hospitalizations, and respiratory exacerbations were secondary outcomes.	At one month, the trial reported Queens College Step Test-estimated VO_2max as an exercise-capacity/cardiorespiratory-fitness outcome, together with PEFR, mMRC dyspnea, and SF-12 quality of life. These outcomes mapped directly to the prespecified primary and secondary outcome domains.	**Eligible**
**Study design**	Individually randomized parallel-group or eligible cluster-randomized trials; quasi-randomized and crossover designs excluded.	Three-arm parallel RCT (60/60/60) using a simple lottery/chit allocation method with equal assignment probability. Allocation concealment was insufficiently documented and participants/assessors were unblinded, but these issues affect RoB 2 judgments rather than randomized-design eligibility.	**Eligible**
**Overall**	All prespecified PICOS components must be satisfied.	No prespecified PICOS exclusion criterion was triggered. The report used the term PTB rather than PTLD in its eligibility wording, but the combination of completed treatment, AFB-negative clinical stability, and baseline post-treatment dyspnea met the review's operational PTLD population definition.	**Include [9]**

Eligibility note: The Sharma report was verified against the published version of record [9]. Its inclusion criteria did not explicitly use the label "PTLD"; however, participants had completed anti-tuberculosis treatment, were AFB-negative and clinically stable, and had ongoing baseline dyspnea. The control arm included routine breathing exercises as part of standard care, but the report did not describe a structured or progressive rehabilitation protocol for controls. These reporting features were therefore not treated as PICOS exclusions. Randomization concealment and lack of blinding remain addressed separately in RoB 2. **Abbreviations:** AFB, acid-fast bacilli; mMRC, modified Medical Research Council; PEFR, peak expiratory flow rate; PICOS, Population, Intervention, Comparator, Outcomes, and Study design; PTLD, post-tuberculosis lung disease; RCT, randomized controlled trial; RoB 2, Cochrane Risk of Bias 2; SF-12, 12-Item Short Form Health Survey; TB, tuberculosis; VO_2max, maximal oxygen uptake.

Supplementary Table S7. Baseline demographic and clinical characteristics of the two randomized controlled trials included in the primary ISWT meta-analysis.

Characteristic	Katagira et al., 2026 [18] Uganda	Akylbekov et al., 2026 [10] Kyrgyzstan
**Randomized sample size**	114 (PR 57; usual care 57)	114 (PR 61; usual care 53)
**Age, years**	43.3 (15.2), mean (SD)	44.5 (15.6), mean (SD)
**Male sex**	65/114 (57.0%)	59/114 (51.8%)
**Time since completion of TB treatment**	65.4 (70.5) months, mean (SD)	Not reported
**Smoking status**	Current 1 (0.9%); former 30 (26.3%); never 83 (72.8%)	Current 29 (25.4%); former 27 (23.7%); never 58 (50.9%)
**HIV status**	30/104 (28.9%) HIV-positive; all HIV-positive participants receiving ART	0 HIV-positive; 57 (50.0%) HIV-negative; 57 (50.0%) status unknown
**Pulmonary function at baseline**	Pre-BD FEV_1: 63.8 (25.1)% predicted; pre-BD FVC: 74.3 (22.5)% predicted	Post-BD FEV_1: 2.59 (0.96) L; post-BD FVC: 3.44 (1.00) L; FEV_1/FVC: 0.75 (0.16)
**Baseline ISWT**	392.6 (131.6) m, mean (SD)	PR: 444.3 m (95% CI 405.0–483.5); control: 474.2 m (95% CI 431.7–516.8)*
**Baseline MRC dyspnea**	Grade 2: 66 (58%); grade 3: 41 (36%); grade 4: 7 (6%)	PR: 2.7 (95% CI 2.5–2.9); control: 2.6 (95% CI 2.4–2.8)*

Characteristic	Katagira et al., 2026 [18] Uganda	Akylbekov et al., 2026 [10] Kyrgyzstan
**Key PTLD eligibility features**	Completed treatment for microbiologically confirmed pulmonary TB; persistent respiratory symptoms; radiologic evidence of TB-related parenchymal damage; MRC dyspnea grade ≥2	Completed TB treatment; confirmed TB-negative; PTLD diagnosed by TB specialists using chest X-ray and sputum analysis; MRC dyspnea grade ≥2

Supplementary Table S8. Structured source-level trustworthiness assessment of Orooj et al. 2023 and Sharma et al. 2026, informed by INSPECT-SR.

Report	Trustworthiness issue	Published observation	Review action / interpretation
**Orooj et al., 2023 [12]**	Internal consistency of baseline 6MWD	Control-group baseline 6MWD differs between published Tables 1 and 2 (352.37 vs 313.0 m).	Treated as an unresolved source-reporting inconsistency; values were not reconstructed. Sensitivity analyses assess the impact of excluding the report.
**Orooj et al., 2023 [12]**	Consistency of reported baseline statistics	The reported baseline between-group P value (P = .17) is difficult to reconcile with the published group summary statistics.	The reported P value was not recalculated or substituted in the review; comparative interpretation is downgraded and the concern is explicitly reported.
**Orooj et al., 2023 [12]**	Plausibility / scaling of pulmonary-function data	Mean FEV_1/FVC values are reported around/above 100, with unclear scaling or units.	FEV_1/FVC values were not reconstructed and are excluded from quantitative synthesis.
**Sharma et al., 2026 [9]**	Control-group data pattern	For all four outcomes in the published table, control-group pre- and post-intervention means and SDs are identical to two decimals.	Treated as an unresolved source-reporting concern; the trial is not used in pooled models and its results are interpreted cautiously.
**Sharma et al., 2026 [9]**	Baseline comparability / VO_2max	Baseline VO_2max differs materially across randomized arms (control 32.80, Lung Flute 26.12, Acapella 23.05 mL/kg/min).	The omnibus post-intervention ANOVA is not interpreted as a causal device-versus-control effect without adequate baseline-adjusted contrasts.
**Sharma et al., 2026 [9]**	Direction and specificity of device effect	Post-intervention VO_2max is 22.62 mL/kg/min in the Lung Flute arm versus 32.80 mL/kg/min in control; the source reports an omnibus ANOVA and Tukey pairwise significance.	The trial is not cited in the Abstract or Conclusions as straightforward evidence of benefit; device-specific claims are qualified.

https://doi.org/10.3390/jcm15218397

**Both reports**	Sensitivity to exclusion	Neither report contributes to the primary adjusted-ISWT meta-analysis. Orooj 2023 contributes to exploratory 6MWD/cross-instrument analyses; Sharma 2026 is narrative only.	Excluding both reports leaves the primary pooled ISWT estimate unchanged. Excluding Orooj 2023 leaves only one trial in the exploratory 6MWD pool, precluding meta-analysis; excluding Sharma 2026 changes no pooled estimate.

Note: Values are reported as presented in the original trial publications. Where reporting formats differed, values were not converted or reconstructed. *For Akylbekov et al., baseline ISWT and MRC values are model-predicted baseline estimates reported in the trial outcome model rather than raw descriptive means. Spirometry is not directly comparable across trials because Katagira et al. reported pre-bronchodilator percent-predicted values, whereas Akylbekov et al. reported post-bronchodilator absolute volumes. Abbreviations: ART, antiretroviral therapy; BD, bronchodilator; CI, confidence interval; FEV_1, forced expiratory volume in one second; FVC, forced vital capacity; HIV, human immunodeficiency virus; ISWT, Incremental Shuttle Walk Test; MRC, Medical Research Council; PR, pulmonary rehabilitation; PTLD, post-tuberculosis lung disease; SD, standard deviation; TB, tuberculosis.

Supplementary Table S9. Deviations from the registered protocol/public record and reasons (PRISMA 2020 Item 24c).

Secondary outcome: inspiratory muscle strength (MIP)	MIP is explicitly listed as a secondary outcome and is included as a separate GRADE/Summary-of-Findings domain. Peripheral muscle strength is retained only as an additional extracted functional outcome.	The earlier manuscript wording omitted MIP from the secondary-outcome list and incorrectly implied that peripheral muscle strength was prespecified.	Corrected in Methods (Outcomes and RoB), Results, Table 8, Table 9, and Conclusions.
Primary quantitative synthesis of exercise capacity	Adjusted between-group ISWT change from Katagira 2026 and Akylbekov 2026 is used as the primary scale-specific meta-analysis.	The registered protocol/public record did not specify the adjusted ISWT model as primary. During revision, the updated evidence set contained two independent RCTs with directly comparable adjusted ISWT change estimates; this approach preserves the same scale and trial-level adjustment and is methodologically preferable to the earlier cross-instrument endpoint SMD.	Timing and rationale are stated explicitly in Methods; the earlier cross-instrument model remains an exploratory sensitivity analysis.

https://doi.org/10.3390/jcm15218397

Planned subgroup analyses	Not performed.	Too few clinically and methodologically comparable RCTs were available within any meaningful subgroup to support credible subgroup estimation.	Stated in Methods and Sensitivity Analyses; no post hoc subgroup effects are claimed.
Planned sensitivity analyses	Only sensitivity analyses feasible with the final evidence set were performed: cross-instrument endpoint analysis, alternative handling of potentially overlapping Orooj reports, and exclusion/qualification of reports with trustworthiness concerns.	Several planned sensitivity analyses required a larger number of comparable trials or data not available in the included reports. The final evidence set also generated unanticipated issues (possible participant overlap and source-level trustworthiness concerns) that required targeted sensitivity analyses.	Reported in Section 3.5 and Supplementary Table S8; limitations are discussed explicitly.
Torres 2025 respiratory-muscle-strength data	The public trial record is acknowledged as specifying MIP/MEP assessment, but no numerical MIP result from the final publication is used in synthesis.	A comparative MIP estimate suitable for extraction was not available in the final published report used for this review.	MIP synthesis therefore relies on the directly extractable Muthukrishnan 2026 estimate; this is stated in Results and Table 9.
Torres 2025 MIP extraction correction	The previous statement that the published Torres trial lacked extractable MIP data is incorrect.	Torres Table 2 reports MIP in both groups before and after rehabilitation. Correct extraction: unadjusted between-arm difference in mean MIP changes +12.1 cmH_2O; published $p=0.266$. Muthukrishnan and Rajadurai 2026 reported +18.6 cmH_2O. Exploratory two-trial pooled change MD +17.75 cmH_2O (95% HK CI −10.05 to 45.56; $I^2 \approx 20.6\%$), newly added post hoc. Torres precision discrepancy unresolved.	Documented correction of extraction/reporting; not prespecified. The analysis is exploratory pending source-statistics clarification and outcome-specific RoB 2 reassessment.
